# Metabolomic signatures in liquid biopsy are associated with overall survival in metastatic melanoma patients treated with immune checkpoint inhibitor therapy

**DOI:** 10.1186/s13046-025-03378-8

**Published:** 2025-04-10

**Authors:** Susan Costantini, Gabriele Madonna, Mariaelena Capone, Elena Di Gennaro, Palmina Bagnara, Federica Renza, Domenico Mallardo, Roberta Affatato, Carlo Vitagliano, Marilena Romanelli, Marilena Tuffanelli, Ester Simeone, Gennaro Ciliberto, Paolo A. Ascierto, Alfredo Budillon

**Affiliations:** 1 Experimental Pharmacology Unit, Istituto Nazionale Tumori—IRCCS—Fondazione G. Pascale, Naples, 80131 Italy; 2 Cancer Immunotherapy and Development Therapeutics Unit, Melanoma, Istituto Nazionale Tumori—IRCCS—Fondazione G. Pascale, Naples, 80131 Italy; 3 IRCCS National Cancer Istituto Regina Elena, Rome, 00144 Italy; 4 Scientific Directorate, Istituto Nazionale Tumori—IRCCS—Fondazione G. Pascale, Naples, 80131 Italy

**Keywords:** Melanoma, Metabolomics, Serum biomarkers, Immune checkpoint inhibitors

## Abstract

**Background:**

Immune checkpoint inhibitors (ICIs), such as anti-Cytotoxic T-Lymphocyte Antigen 4(CTLA-4) and anti-Programmed cell death protein 1 (PD-1) agents, have improved the prognosis of patients with metastatic melanoma. However, a proportion of patients develop resistance to these treatments, leading to a poor prognosis. Therefore, identifying potential non invasive and easy to measure biomarkers is crucial for guiding treatment strategies in patients with metastatic melanoma.

**Methods:**

A retrospective single-center study was conducted involving patients with metastatic stage IV melanoma who received first-line treatment with anti-CTL4 and/or anti-PD-1 agents. The patients were categorized into two groups on the basis of their 1-year overall survival (OS): those with good outcomes (long-term OS ≥ 1 year) and those with poor outcomes (short-term OS < 1 year). Peripheral metabolomics was performed using baseline sera from 132 patients via 600 MHz Nuclear Magnetic Resonance (NMR) spectroscopy. Enriched functional analysis was conducted to identify the metabolic pathways in which significant metabolites were involved.

**Results:**

Sparse partial least squares discriminant analysis (sPLS-DA) and loading plots obtained by analyzing the metabolomics profiles of samples collected before ICI treatment revealed significantly different levels of metabolites between the two groups (long-term OS vs. short-term OS). Specifically, lactate, tryptophan and valine significantly predicted the OS of the whole study population subjected to ICI immunotherapy; alanine, asparagine, glutathione, histidine, isoleucine and phenylalanine significantly predicted the OS of patients treated with ipilimumab; glucose, glutamine, histidine and proline significantly predicted the OS of patients treated with nivolumab; and lactate, lysine and proline significantly predicted the OS of patients treated with ipilimumab plus nivolumab. Notably, tryptophan levels were correlated with treatment response in the overall patient group, whereas histidine and lactate levels were associated with response in patients treated with ipilimumab and with ipilimumab plus nivolumab, respectively. Interestingly, higher pretreatment levels of histidine were commonly found in long-term OS subgroups of patients treated with ipilimumab, nivolumab or ipilimumab plus nivolumab. Interestingly, considering only those metabolites that predict OS after univariate analysis, higher histidine, and lower lactate and proline levels resulted as associated with favorable OS in at least two patient cohorts.

**Conclusions:**

Overall, this exploratory liquid biopsy study revealed a strong correlation between the pretreatment levels of some metabolites and the OS of patients with metastatic stage IV melanoma treated with anti-CTL4 and/or anti-PD-1 antibodies in the first-line setting and revealed the potential of these molecules to predict outcomes and define personalized management and treatment strategies.

**Supplementary Information:**

The online version contains supplementary material available at 10.1186/s13046-025-03378-8.

## Background

Metastatic melanoma (MM) is one of the deadliest cutaneous neoplasms and its incidence has increased worldwide, however the introduction of immune checkpoint inhibitors, such as CTLA-4 and PD-1 targeting antibodies, in the therapeutic management of MM changed completely the clinical history of MM patients significantly improving survival also in the metastatic setting [1]. Although ipilimumab was the first single agent ICI approved by the Food and Drug Administration (FDA) for the treatment of MM [2], different studies provided convincing evidence that anti–PD-1 agents are superior to ipilimumab in terms of OS and progression free survival (PFS) in advanced melanoma patients, for whom durable benefits continue to be observed after a recent 10-year follow-up [3, 4]. Currently, PD-1 inhibitors are considered the standard of care as adjuvant therapy in high-risk resected stage III or in metastatic stage IV melanoma patients, and tumor regression and long-term durable cancer control are possible in a high proportion of patients, compared with < 10% before the introduction of immunotherapy [5]. More recently, several studies revealed significant improvements in objective response rate (ORR), PFS, and OS with nivolumab plus ipilimumab compared with single agents in patients with advanced melanoma [4, 6]. These data were confirmed via long-term analyses, which revealed a median OS (minimum follow-up, 10 years) of 71.9 months in the nivolumab plus ipilimumab combination group compared with 36.9 and 19.9 months in the nivolumab and ipilimumab groups, respectively. An improvement in terms of the 10-year OS rate of 52% was also reported in patients with BRAF-mutant tumors treated with nivolumab plus ipilimumab versus 37% and 25% in the nivolumab and ipilimumab groups, respectively. Similarly, for BRAF-wild-type tumors, a 39% 10-year OS rate was achieved after combination treatment, compared with 37% for nivolumab and 17% for ipilimumab alone [4].

However, unmet needs still exist because many patients fail to benefit from ICI treatments or develop primary or acquired resistance to these treatments after initial response. Therefore, there is a pressing need to identify patients with low probability to benefit from ICI treatment and also to find novel therapeutic approach to target the mechanism of resistance. Numerous studies have been conducted on potential circulating biomarkers to evaluate the efficacy of ICIs [7]. Analyses of melanoma patients treated with ipilimumab revealed that improved PFS and OS were associated with a low neutrophil‒lymphocyte ratio (NLR), low absolute neutrophil count, low frequency of myelogenous suppressor cells, low absolute monocyte count, high eosinophil count, high lymphocyte frequency and high frequency of forkhead box P3 (FoxP3) + Treg cells [8]. Moreover, reports for patients with melanoma treated with PD-1 inhibitor have shown that an increased NLR is associated with a worse tumor response and worse outcome [9]. On the other hand, low baseline lactate dehydrogenase (LDH) levels and high relative/absolute eosinophil counts are associated with prolonged OS in patients with melanoma treated with PD-1 inhibitor [10]. More recently, in patients with unresectable/metastatic melanoma (stage IIIb-IV) treated with anti-PD-1 therapy in the first-line setting, a high baseline NLR was associated with a worse prognosis and higher LDH levels. Additionally, the increased expression of CD39, a gene positively associated with the NLR, was found to be correlated with the expression of transforming growth factor beta (TGFβ)2, a marker of N2 neutrophils with immunosuppressive activity. These data suggest that a high NLR is associated with an increased neutrophil population polarized toward the N2 phenotype, which may underlie the negative prognostic role of the NLR [11].

Since metabolism contributes to immune cell activation and immune function, with immune cells adopting specific metabolic programs [12], and the modulation of metabolic processes in immune cells may enhance immune responses [13], the evaluation of metabolomic biomarkers in liquid biopsy emerged in recent years as a powerful prognostic and/or predictive tool to be used in cancer patients undergoing immunotherapy [14, 15]. Indeed, the application of metabolomic profiling to biological fluids represents a powerful and reliable approach for identifying novel biomarkers [16, 17]. Specifically, NMR spectroscopy represents the only nondestructive technique able to rapidly identify and quantify complex mixtures of metabolites in small samples, and its use is increasing for successful patient stratification in various diseases, including cancer [18, 19].

In this study, we evaluated serum metabolomics using an NMR approach in patients with metastatic stage IV melanoma at baseline prior to treatment with different ICIs (ipilimumab, nivolumab and ipilimumab plus nivolumab) in the first-line setting. Our aim was to identify metabolites and metabolic pathways that could predict OS in response to ICIs and explore potential personalized treatment strategies.

## Methods

### Patient characteristics

This retrospective single-center study included a total of 132 patients (male, *n* = 72; female, *n* = 60) aged > 18 years with metastatic stage IV melanoma (Table 1**)**. Samples were collected between 2018 and 2022 at the Melanoma, Cancer Immunotherapy and Innovative Therapies Unit of the National Cancer Institute of Naples “G. Pascale Foundation”, Italy (protocol code: 33/17 OSS, date of approval: 10/1/2018). Written informed consent was obtained from all patients in accordance with the Declaration of Helsinki for the use of human biological samples for research purposes.

**Table 1:** Clinical data of patients

	All patients			Patients treated with ipilimumab in first line setting			Patients treated with nivolumab in first line setting			Patients treated with ipilimumab plus nivolumab in first line setting		
	Training set	Validation set	*p*-value	Training set	Validation set	*p*-value	Training set	Validation set	*p*-value	Training set	Validation set	*p*-value
**Number of patients (*n*** **= 132)**	64	68		21	27		18	23		25	18	
**Gender** * n*												
Male	37	35	0.46	11	16	0.63	10	13	0.95	16	6	0.05
Female	27	33		10	11		8	10		9	12	
**Age** * n*												
<=50 years	16	21	0.45	7	6	0.39	3	7	0.31	6	8	0.16
> 50	48	47		14	21		15	16		19	10	
**BRAF** * n*												
WT	39	41	0.99	13	21	0.23	13	14	0.71	13	6	0.14
Mutation	23	25		8	6		5	7		10	12	
Unknown	2	2						2		2		
**LDH** * n*												
Normal	32	40	0.059	15	14	0.06	7	11	0.24	10	15	0.01*
Elevated	18	23		2	9		3	12		13	2	
Unknown	14	5		4	4		8			2	1	
**Brain metastates** * n*												
Yes	30	31	0.62	2	7	0.13	3	7	0.31	25	17	0.24
No	34	36		19	19		15	16			1	
Unknown	0	1			1							
**Distant MTX at start of treatment** * n*												
Soft tissue only	11	11	0.33	5	6	0.09	6	2	0.03*		3	0.10
Lung+/-soft tissue	12	12			4		5	3		7	5	
Visceral+/- soft tissue +/- lung	40	39		15	12		7	18		18	9	
None	1	6		1	5						1	
**Response** * n*												
CR	6	5	0.84	3	1	0.27	1	2	0.42	3	2	0.35
PR	13	18		1	2		5	7		6	9	
SD	14	13		8	6		3	5		4	2	
PD	31	32		9	18		9	9		12	5	
**OS** * n*												
long-term	36	39	0.90	14	17	0.79	7	12	0.40	15	10	0.77
short-term	28	29		7	10		11	11		10	8	

A total of 80 patients out of 132 (60.6%) had wild-type BRAF, 48 patients (36.4%) had BRAF V600 mutations, 45 patients (34.1%) had brain metastases, and 41 (31.1%) had elevated lactate dehydrogenase levels (LDH > 480 international units [IU]/liter).

Ipilimumab (3 mg/kg) was administered to 48 patients every three weeks for a total of four cycles or until disease progression or unacceptable toxicity occurred. Nivolumab (3 mg/kg) was administered to 41 patients every 2 weeks until disease progression or unacceptable toxicity occurred. Ipilimumab (3 mg/kg) plus nivolumab (1 mg/kg) was administered every 3 weeks for four doses, and then, only nivolumab (480 mg) was administered every 4 weeks to 43 patients until disease progression or unacceptable toxicity appeared. All drugs were administered as first-line treatments. Tumor response was evaluated at 12 weeks and then every 12 weeks until progression or discontinuation of treatment according to the Response Evaluation Criteria In Solid Tumors (RECIST) criteria; tumor response was radiologically evaluated and classified as complete response (CR), partial response (PR), stable disease (SD), or progressive disease (PD). The disease control rate (DCR) is defined as the sum of the CR, PR and SD rates. The overall response rate (ORR) to first-line ipilimumab was 14.6% (7/48): 4 patients achieved CR, and 3 achieved PR. The DCR to ipilimumab was 43.8% (21/48: 4 patients with CR, 3 with PR and 14 with SD). A total of 56.2% of patients (27/48) presented with PD.

The ORR to first-line nivolumab was 36.6% (15/41): 3 patients achieved CR, and 12 patients achieved PR. The DCR to first-line nivolumab was 56.1% (23/41): 3 patients achieved CR, 12 achieved PR, and 8 achieved SD; 43.9% of patients (18/41) presented with PD.

The ORR to first-line ipilimumab plus nivolumab was 46.5% (20/43): 5 patients achieved CR, and 15 achieved PR. The DCR to first-line ipilimumab plus nivolumab was 60.5% (26/43): 5 patients achieved CR, 15 achieved PR, and 6 achieved SD; 39.5% of patients (17/43) presented with PD.

### Serum ^1^H NMR spectroscopy

Samples for NMR analysis were prepared by mixing 330 µL of serum with 300 µL of phosphate-buffered saline (PBS) (containing 10% v/v D_2_O) and 70 µL of reference standard D_2_O solution containing 0.1 mM sodium 3-trimethylsilyl [2,2,3,3-2H4] propionate (TSP). All of the spectra were recorded using a Bruker Avance 600 NMR spectrometer operated at a 599.97 MHz ^1^H resonance frequency and equipped with a TCI cryoprobe. A standard Carr–Purcell–Meiboom–Gill (CPMG) presaturation pulse sequence was used to attenuate the broad NMR signals from slowly tumbling molecules due to proteins and lipids and to suppress the water peaks using the parameters reported in our recent paper [17, 20]. In our experiment, the data points were acquired using 256 transients.

### NMR data processing and statistical analysis

The ^1^H NMR spectra acquired from all the serum samples were manually phased, baseline-corrected and calibrated with the CH_3_ resonance of TSP at 0 ppm as a reference. The spectral 0.50–8.60 ppm region of the ^1^H-NMR spectra was integrated into 0.04 ppm buckets, excluding the water resonance region (4.5–5.2 ppm), via the AMIX 4.0.2 package (Bruker, Biospin, Germany). The bucketed regions were normalized to the total spectrum area using Pareto scaling using MetaboAnalyst v5.0 [21].

To explain the maximum separation between the defined class samples in the data, the sparse partial least squares-discriminant analysis (sPLS-DA) algorithm was applied. Score and loading plots were used to assess the role of NMR signals in the classification models and, hence, to identify the top 10 significant NMR signals. Metabolite assignment was performed using reference metabolite spectra from the Human Metabolome Database (HMDB) [22].

Receiver operating characteristic (ROC) curves were generated for significant metabolites using the Biomarker Analysis tool in Metaboanalyst v5.0 [21]. The area under the curve (AUC) was used to assess accuracy.

The differences in terms of OS between groups with higher and lower cutoff values were tested by the log-rank test and represented by Kaplan–Meier curves using MedCalc software (https://www.medcalc.org).

A Cox regression model was used to assess the role of the cutoff values for metabolites in predicting OS. Hazard ratios (HRs) were derived from the Cox regression analysis, and 95% confidence intervals (95% CIs) were calculated using the proportional hazard model. On the basis of the metabolite parameter cutoff values, univariate and multivariate analyses were performed using MedCalc software (https://www.medcalc.org). Univariate analysis was used to assess the correlations of baseline patient characteristics and metabolites with OS. A multivariate analysis was conducted via the backward elimination of factors with a p value (P) less than 0.05 in the univariate analysis.

The chi-square test was used to study associations among categorical variables. In all the statistical tests, a p value less than 0.05 was considered significant.

Finally, biomarker analyses were performed on the basis of ROC curves for multiple metabolites via the support vector machine (SVM) algorithm with the module “Biomarker Analysis” in Metaboanalyst 5.0 [21], as previously described [17, 20].

### Enriched pathway analysis of significant metabolites

Enriched pathway analysis of the modulated metabolites was performed using Metaboanalyst v5.0 [21] with the following parameters: variable importance plot (VIP) values > 1 in class discrimination; correlation values > 0.8; *Homo sapiens* pathway library; and the Fisher exact test for overrepresentation and relative betweenness centrality in topological analyses.

## Results

### Baseline serum metabolic profile can discriminate metastatic melanoma patients with long-term versus short-term survival treated with first-line immune checkpoint inhibitors-based immunotherapy

Sera from a cohort of patients with metastatic stage IV melanoma were collected at baseline prior to first-line treatment with ipilimumab, nivolumab or the combination of ipilimumab plus nivolumab. The samples were subdivided into training and validation sets and analyzed on the basis of 1-year OS. Patients were categorized into two groups: those with a good outcome (long-term OS) with an OS ≥ 1 year and those with a poor outcome (short-term OS), with an OS < 1 year (Table 1).

To investigate whether serum metabolites differ between the 36 long-term OS patients and the 28 short-term OS patients and to evaluate the potential of serum metabolomic profiling for predicting patient outcomes, we first used an ^1^H-NMR approach to analyze all the collected pretreatment sera from patients independent of the ICI treatment received.

**Fig. 1:**
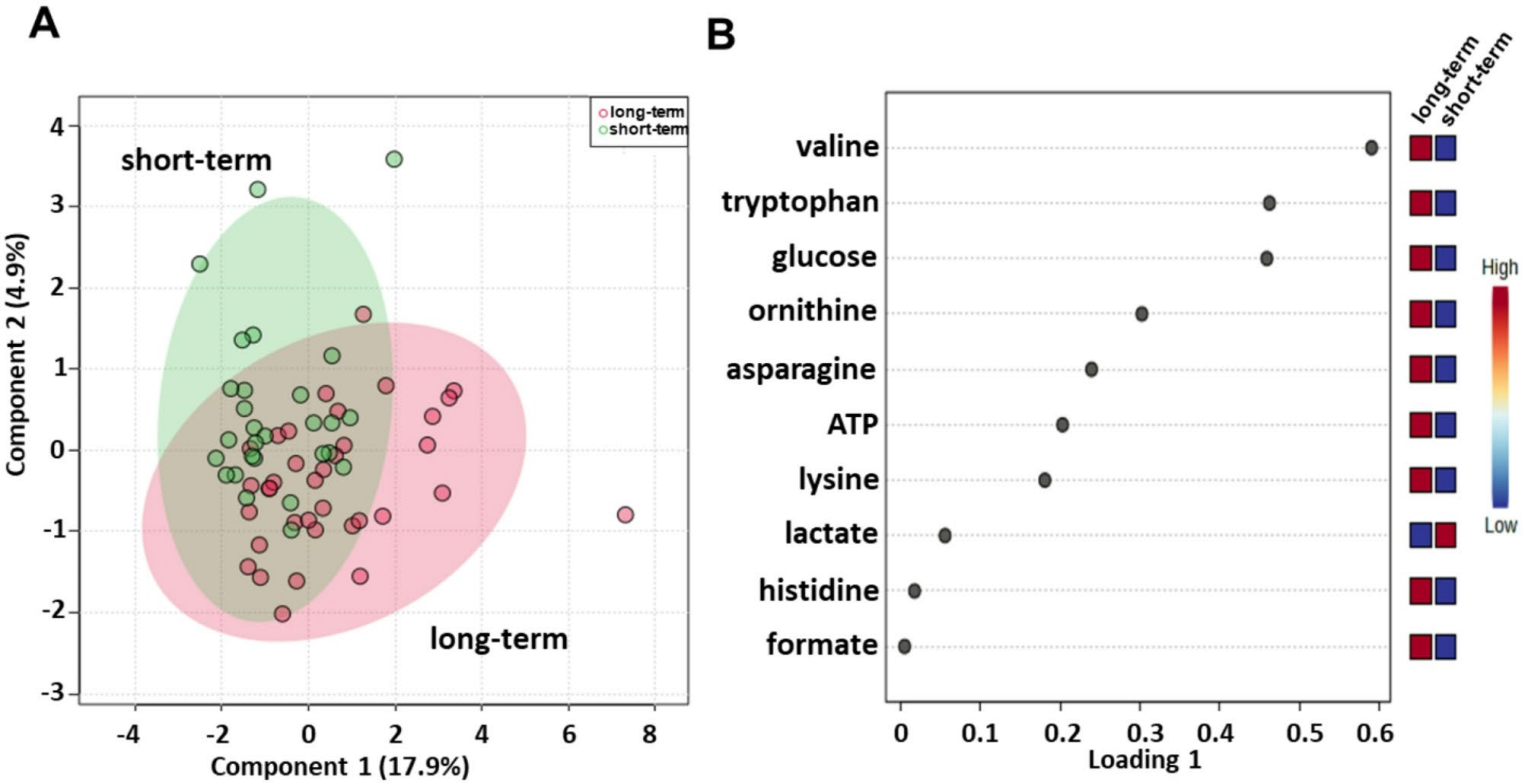
Score plot (**A**) and loading plot (**B**) related to metabolomic profiling of the sera of all metastatic melanoma patients treated with immunotherapy at first-line enrolled in training set, and subdivided accordingly to OS in good (long-term; OS ≥ 1 year) and poor (short-term; OS < 1 year) outcome

Sparse partial least squares discrimination analysis (sPLS-DA) (22.8% of the total variance) revealed that the long-term compared with short-term serum metabolomics profiles grouped into two different clusters (Fig. 1A). PLS loading analysis was then conducted to identify the metabolites responsible for class separation. As shown in Fig. 1B, the loading plot revealed lower levels of lactate and higher levels of asparagine, adenosine triphosphate (ATP), formate, glucose, histidine, lysine, ornithine, tryptophan and valine in the long-term compared to the short-term OS group.

Moreover, these metabolites were used to perform metabolite set enrichment analysis and to identify several different metabolic pathways in which they are involved (Fig. S1; Table S1). Specifically, biotin metabolism; ammonia recycling; lactose degradation; gluconeogenesis; trehalose degradation; spermidine and spermine biosynthesis; lactose synthesis; the Warburg effect; tryptophan metabolism; the transfer of acetyl groups into mitochondria; glycolysis; the urea cycle; folate metabolism; methylhistidine metabolism; and aspartate metabolism emerged as pathways that discriminated the serum metabolic profiles of the two groups.

A validation set was also tested, confirming a clear separation in the score plot between 39 long-term OS and 29 short-term OS patients (Fig. S2A) and same metabolites identified in the training set correlating with OS (Fig. S2B).

**Fig. 2:**
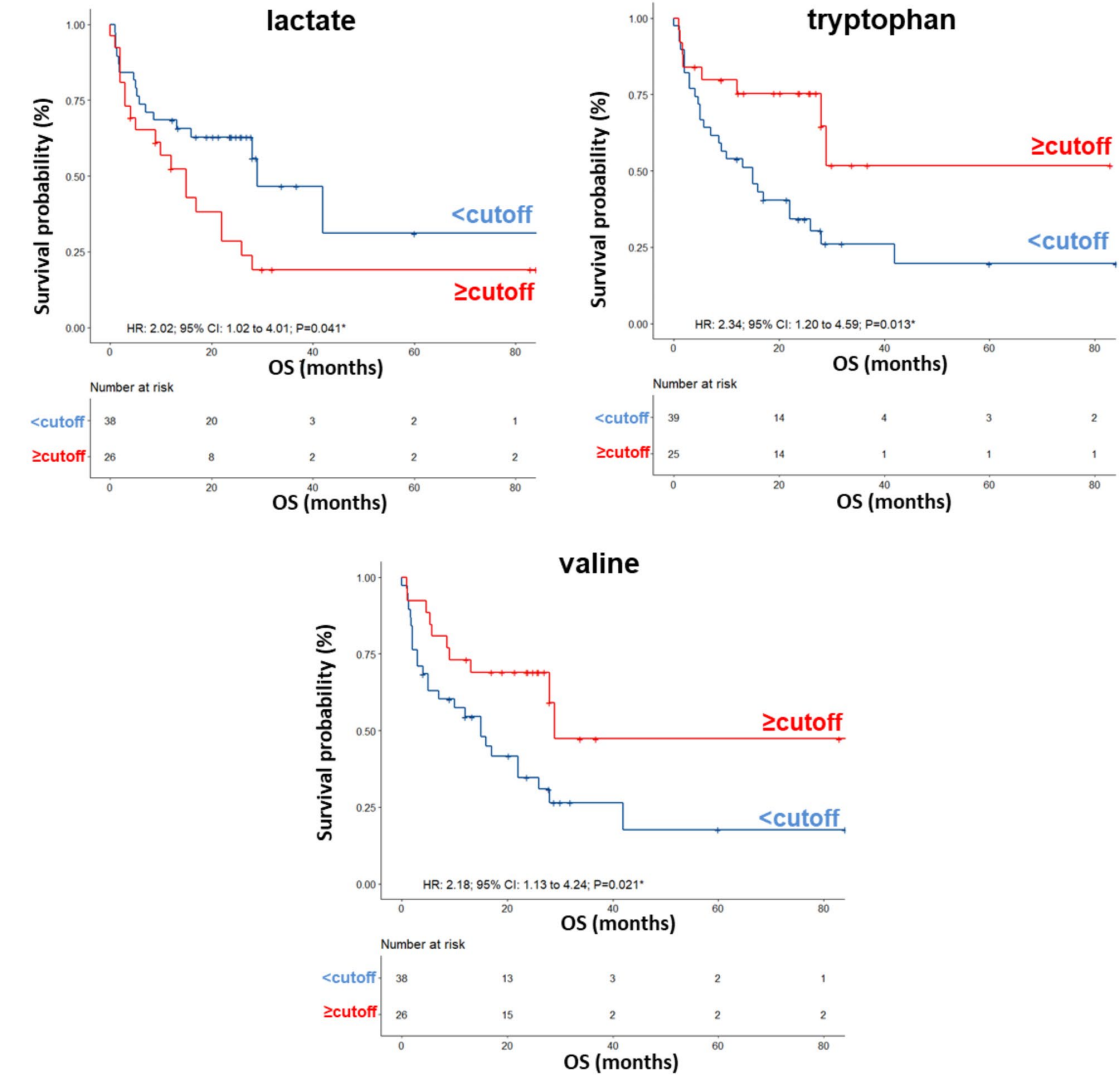
Kaplan–Meier curves of OS accordingly to lactate, tryptophan and valine in the sera from metastatic melanoma patients treated with immunotherapy at first-line enrolled in training set (log-rank p values are reported). * symbols indicate p values < 0.05

Next, to determine the optimal cutoff value for the metabolites selected by sPLS-DA, we performed ROC curve analysis, which revealed AUC values ranging between 0.60 and 0.66. Although not very high, these values were considered acceptable (Fig. S3). Using the metabolite parameter cutoff values, we conducted univariate and multivariate analyses, on the training set, to identify metabolites potentially associated with OS. Univariate analysis revealed no significant associations between OS and sex, age, BRAF status, LDH, or brain metastases (Table 2). Among the metabolites, as also shown by the Kaplan − Meier (K-M) survival curves, only lower levels (< cutoff) of lactate (HR: 2.02; 95% CI: 1.02–4.01; *P* = 0.041), or higher levels (≥ cutoff) of tryptophan (HR: 2.34; 95% CI: 1.20–4.59; *P* = 0.013), and valine (HR: 2.18; 95% CI: 1.13–4.24; *P* = 0.021), evaluated before first-line treatment, were correlated with a more favorable OS (Fig. 2). In the multivariate analysis, tryptophan was the only metabolite that significantly predicted long-term OS (Table 2).

**Table 2:** Univariate and multivariate analyses of baseline patient characteristics and metabolites for OS in all patients

	Univariate	Multivariate
	HR (95% CI) *P* value	HR (95% CI) *P* value
**Patients characteristics**		
**Gender** (M vs. F)	1.50(0.76–2.96) *P* = 0.24	-
**Age**,** years** ( < = 50 vs. > 50)	1.34(0.64–2.80) *P* = 0.44	-
**BRAF status** (mut vs. wt)	1.23(0.61–2.45) *P* = 0.56	-
**LDH**,** IU/L** (≥ 480 vs. < 480)	1.28(0.54–3.02) *P* = 0.57	-
**Brain metastates**(no vs. yes)	1.01(0.51–1.98) *P* = 0.98	-
**Metabolites(nps)**		
**Asparagine level** (<-0.97 vs. ≥-0.97)	1.75(0.90–3.41) *P* = 0.097	-
**ATP level** (<-0.0269 vs. ≥-0.0269)	1.37(0.68–2.76) *P* = 0.38	-
**Formate level** (< 0.0883 vs. ≥ 0.0883)	1.54(0.79–2.99) *P* = 0.20	-
**Glucose level** (<-1.28 vs. ≥ 1.28)	1.87(0.96–3.63) *P* = 0.06	-
**Histidine level** (<-1.49 vs. ≥-1.49)	1.61(0.83–3.13) *P* = 0.16	-
**Lactate level** (≥ 4.62 vs. < 4.62)	2.02(1.02–4.01) ***P*** = 0.041*	1.40(0.65–2.99) *P* = 0.38
**Lysine level** (<-1.19 vs. ≥-1.19)	1.39(0.70–2.75) *P* = 0.35	-
**Ornithine level** (<-0.391 vs. ≥-0.391)	1.38(0.67–2.86) *P* = 0.38	-
**Tryptophan level** (< 0.0555 vs. ≥ 0.0555)	2.34(1.20–4.59) ***P*** = 0.013*	1.38(1.17–1.84) ***P*** = 0.017*
**Valine level** (< 0.602 vs. ≥ 0.602)	2.18(1.13–4.24) ***P*** = 0.021*	0.73(0.29–1.85) *P* = 0.51

HR: hazard ratio; CI: confidence interval; nps: normalized values of the proton signals. M: male; F: female; mut: mutant; wt: wild type; LDH: lactate dehydrogenase; IU/L: International Units per Liter; Significant p-values < 0.05 are reported in bold

Interestingly by analyzing the correlation between the serum levels of metabolites and patient response to ICIs, measured as DCR, we found again association between high levels of tryptophan and good response to ICI immunotherapy (OR: 4.11; 95% CI: 1.39–12.19; *P* = 0.0088) (Table 3).

**Table 3:** Response to treatment related to the levels of the significant metabolites for all patients

	CR	PR	SD	PD	DCR(CR + PR + SD)	*p*-value; OR(95%CI)^§^
**Patient number**	6	13	14	31	33	
**Asparagine level** (<-0.97)		5	8	17	13	0.22
**Asparagine level** (≥-0.97)	6	8	6	14	20	
**ATP level** (<-0.0269)	3	4	8	17	15	0.45
**ATP level** (≥-0.0269)	3	9	6	14	18	
**Formate level** (< 0.0883)	3	8	6	12	17	0.31
**Formate level** (≥ 0.0883)	3	5	8	19	16	
**Glucose level** (<-1.28)	2	4	8	19	14	0.13
**Glucose level** (≥ 1.28)	4	9	6	12	19	
**Histidine level** (<-1.49)	4	7	5	22	16	0.067
**Histidine level** (≥-1.49)	2	6	9	9	17	
**Lactate level** (< 4.62)	5	10	8	15	23	0.082
**Lactate level** (≥ 4.62)	1	3	6	16	10	
**Lysine level** (<-1.19)	1	6	6	12	13	0.96
**Lysine level** (≥-1.19)	5	7	8	19	20	
**Ornithine level** (<-0.391)	2	5	5	10	12	0.73
**Ornithine level** (≥-0.391)	4	8	9	21	21	
**Tryptophan level** (< 0.0555)	2	8	5	24	15	0.0088**; 4.11(1.39–12.19)
**Tryptophan level** (≥ 0.0555)	4	5	9	7	18	
**Valine level** (< 0.602)	2	7	8	21	17	0.18
**Valine level** (≥ 0.602)	4	6	6	10	16	

CR: complete response; PR: partial response; SD: stable response; PD: progression disease; DCR: disease control rate; ^§^Association between the response and the metabolites levels was evaluated by chi square test. When the association is statistically significant (p-value < 0.05), we reported also odds ratio (OR) and 95% confidence interval (CI)

### Identification of baseline serum metabolites that can predict the outcome of patients treated with ipilimumab in the first-line setting

Since the selected cohort of melanoma patients that we have evaluated received three different treatments in the first-line setting (ipilimumab or nivolumab or ipilimumab plus nivolumab), we decided to repeat the previous analyses on the three separate treatment groups to determine which metabolites identified in the overall cohort remained significant and whether there were specific metabolites associated with each treatment group.

**Fig. 3:**
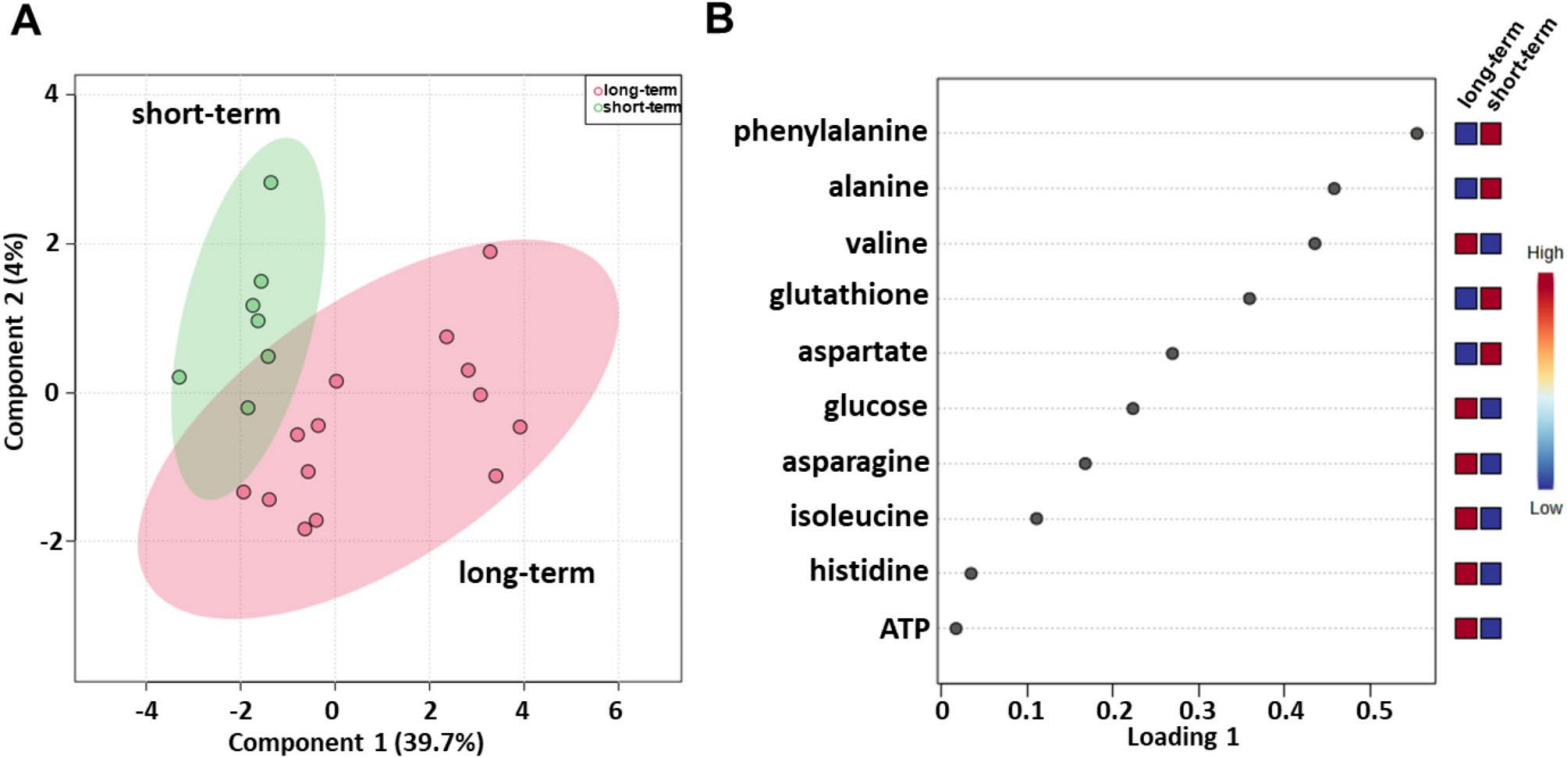
Score plot (**A**) and loading plot (**B**) related to metabolomic profiling of the sera of metastatic melanoma patients treated with ipilimumab at first-line enrolled in training set, and subdivided accordingly to OS in good (long-term; OS ≥ 1 year) and poor (short-term; OS < 1 year) outcome

Serum metabolomic profiling was evaluated using baseline samples from a cohort of patients treated with first-line ipilimumab (Table 1), consisting of 21 patients, i.e., 14 long-term and 7 short-term OS patients. sPLS-DA (44.6% of the total variance) revealed that the metabolomic profiles of the long-term OS group clustered separately from those of the short-term OS group also in this cohort of patients (Fig. 3A). As shown in Fig. 3B, the loading plot revealed lower levels of alanine, aspartate, glutathione and phenylalanine and higher levels of asparagine, ATP, glucose, histidine, isoleucine and valine in the long-term OS group.

Metabolite set enrichment analysis revealed that ammonia recycling; glutamate metabolism; glutathione metabolism; urea cycle; lactose degradation; aspartate metabolism; glucose‒alanine cycle; alanine metabolism; lactose synthesis; valine, leucine and isoleucine degradation; transfer of acetyl groups into mitochondria; glycolysis; phenylalanine and tyrosine metabolism; selenoamino acid metabolism; methylhistidine metabolism; beta-alanine metabolism; and gluconeogenesis were pathways that discriminated the serum metabolic profiles of the two groups (Fig. S4; Table S2).

**Fig. 4:**
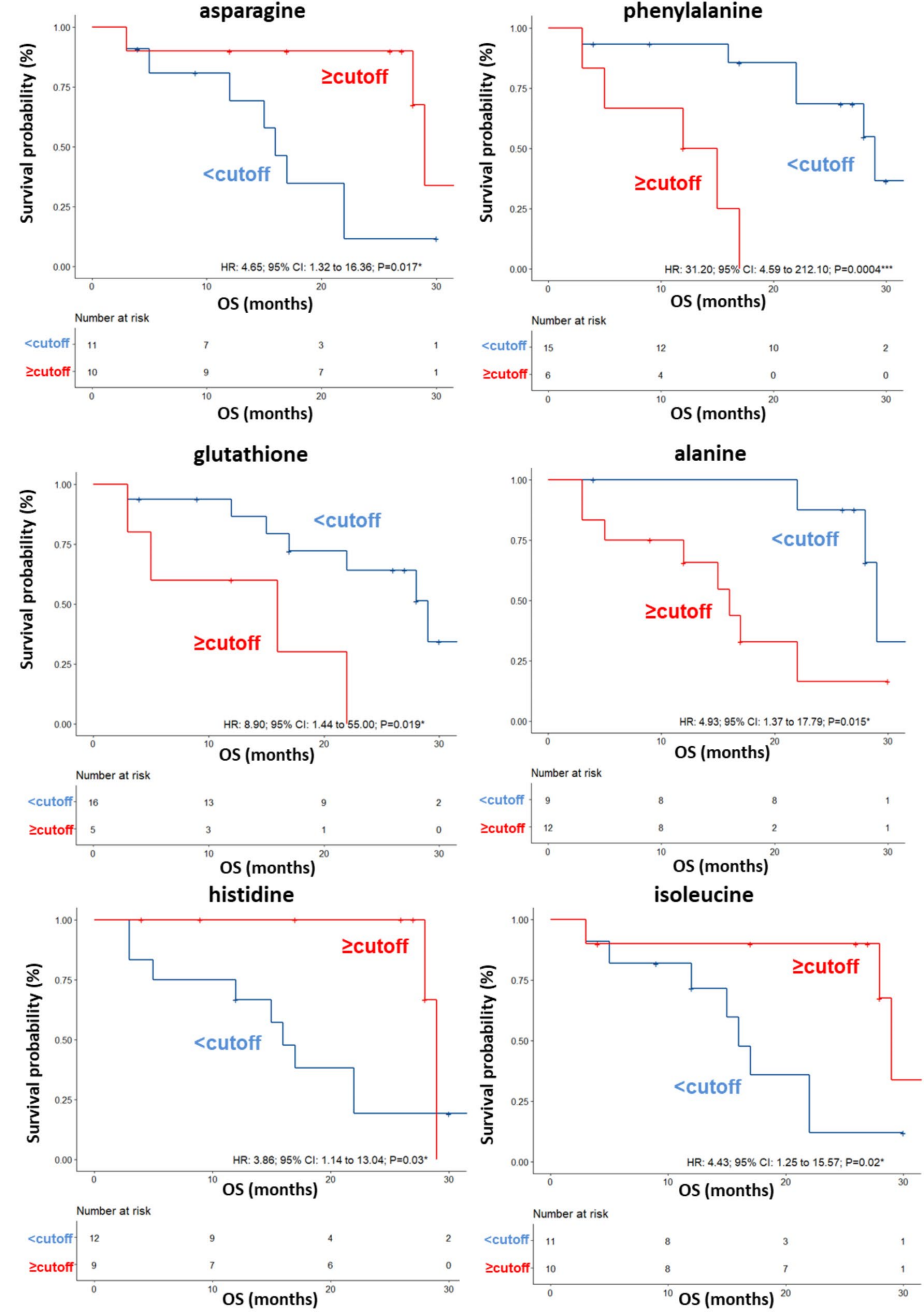
Kaplan–Meier curves of OS accordingly to asparagine, phenylalanine, glutathione, alanine, histidine and isoleucine in the sera of metastatic melanoma patients treated with ipilimumab at first-line enrolled in training set (log-rank p values are reported). * and *** symbols indicate p values < 0.05 and < 0.0001, respectively

A validation set was also tested, confirming a clear separation in the score plot between 17 long-term OS patients and 10 short-term OS patients (Fig. S5A) and same metabolites identified in the training set correlating with OS (Fig. S5). ROC curve analysis was performed (AUC values ranging between 0.75 and 0.908) (Fig. S6) and again the univariate analysis did not reveal any significant associations between OS and anagraphical, clinical or biochemical parameter reported in Table 4, for this cohort of patients. Among the metabolites, as also shown by the K-M curves, lower levels (< cutoff) of alanine (HR: 4.93; 95% CI: 1.37–17.79; *P* = 0.015), glutathione (HR: 8.90; 95% CI: 1.44–55; *P* = 0.019) and phenylalanine (HR: 31.20; 95% CI: 4.59–212.10; *P* = 0.0004) as well as higher levels (≥ cutoff) of asparagine (HR: 4.65; 95% CI: 1.32–16.36; *P* = 0.017), histidine (HR: 3.86; 95% CI: 1.14–13.04; *P* = 0.03), isoleucine (HR: 4.43; 95% CI: 1.25–15.57; *P* = 0.02) evaluated before first-line treatment, correlated with a more favorable OS (Table 4; Fig. 4). In the multivariate analysis, glutathione and phenylalanine were the only parameters that significantly predicted long-term OS (Table 4). We also found that DCR to ipilimumab was associated with high serum levels of histidine (OR: 11.20; 95% CI: 1.04–120.4; *P* = 0.027). (Table 5).

**Table 4:** Univariate and multivariate analyses of baseline patient characteristics and metabolites for OS in patients treated with ipilimumab at first line

	Univariate	Multivariate
	HR (95% CI) *P* value	HR (95% CI) *P* value
**Patients characteristics**		
**Gender** (M vs. F)	3.24(0.92–11.38) *P* = 0.07	-
**Age**,** years** ( < = 50 vs. > 50)	2.21(0.57–8.50) *P* = 0.25	-
**BRAF status** (mut vs. wt)	1.65(0.46–5.98) *P* = 0.44	-
**LDH**,** IU/L** (≥ 480 vs. < 480)	19.02(0.37-983.33) *P* = 0.14	-
**Brain metastates**(no vs. yes)	1.77(0.34–9.16) *P* = 0.49	-
**Metabolites(nps)**		
**Alanine level** (≥ 0.813 vs. < 0.813)	4.93(1.37–17.79) ***P*** = 0.015*	1.87(0.040–87.87) *P* = 0.75
**Asparagine level** (<-0.175vs ≥-0.175)	4.65(1.32–16.36) ***P*** = 0.017*	1.04(0.040–4.28) *P* = 0.18
**Aspartate level** (≥ 2.1 vs. < 2.1)	2.88(0.72–11.43) *P* = 0.13	-
**ATP level** (<-0.0161 vs. ≥-0.0161)	1.98(0.53–7.33) *P* = 0.30	-
**Glucose level** (<-1.01 vs. ≥-1.01)	1.99(0.54–7.36) *P* = 0.30	-
**Glutathione level** (≥ 0.712 vs. < 0.712)	8.90(1.44-55.00) ***P*** = 0.019*	4.08(1.91–18.35) ***P*** = 0.047*
**Histidine level** (<-2.93 vs. ≥-2.93)	3.86(1.14–13.04)***P*** = 0.03*	1.57(0.047–6.99) *P* = 0.66
**Isoleucine level**(<-3.01 vs. ≥-3.01)	4.43(1.25–15.57) ***P*** = 0.02*	1.87(0.077–9.88) *P* = 0.24
**Phenylalanine level** (≥ 0.342 vs. < 0.342)	31.20(4.59–212.10) ***P*** = 0.0004***	12.87(2.05–80.91) ***P*** = 0.0065**
**Valine level** (<-0.677 vs. ≥-0.677)	2.32(0.66–8.24) *P* = 0.19	-

HR: hazard ratio; CI: confidence interval; nps: normalized values of the proton signals. M: male; F: female; mut: mutant; wt: wild type; LDH: lactate dehydrogenase; IU/L: International Units per Liter; Significant p-values < 0.05 are reported in bold

**Table 5:** Response to treatment related to the levels of the significant metabolites for patients treated with ipilimumab at first-line

	CR	PR	SD	PD	DCR(CR + PR + SD)	*p*-value; OR(95%CI)^§^
**Patient number**	3	1	8	9	12	
**Alanine level** (< 0.813)	2	1	3	3	6	0.44
**Alanine level** (≥ 0.813)	1		5	6	6	
**Asparagine level** (<-0.175)			5	6	5	0.26
**Asparagine level** (≥-0.175)	3	1	3	3	7	
**Aspartate level** (< 2.1)	3	1	5	5	9	0.35
**Aspartate level** (≥ 2.1)			3	4	3	
**ATP level** (<-0.0161)	2		2	4	4	0.61
**ATP level** (≥-0.0161)	1	1	6	5	8	
**Glucose level** (<-1.01)	1		3	5	4	0.30
**Glucose level**(≥-1.01)	2	1	5	4	8	
**Glutathione level**(< 0.712)	2	1	7	6	10	0.37
**Glutathione level**(≥ 0.712)	1		1	3	2	
**Histidine level** (<-2.93)	1	1	3	8	5	0.027*;11.20(1.04–120.4)
**Histidine level** (≥-2.93)	2		5	1	7	
**Isoleucine level** (<-3.01)	1		4	6	5	0.26
**Isoleucine level** (≥-3.01)	2	1	4	3	7	
**Phenylalanine level** (< 0.342)	2	1	7	5	10	0.16
**Phenylalanine level** (≥ 0.342)	1		1	4	2	
**Valine level** (<-0.677)	1		3	5	4	0.31
**Valine level** (≥-0.677)	2	1	5	4	8	

CR: complete response; PR: partial response; SD: stable response; PD: progression disease; DCR: disease control rate; ^§^Association between the response and the metabolites levels was evaluated by chi square test. When the association is statistically significant (p-value < 0.05), we reported also odds ratio (OR) and 95% confidence interval (CI)

### Identification of baseline serum metabolites that can predict the outcome of patients treated with nivolumab in the first-line setting

**Fig. 5:**
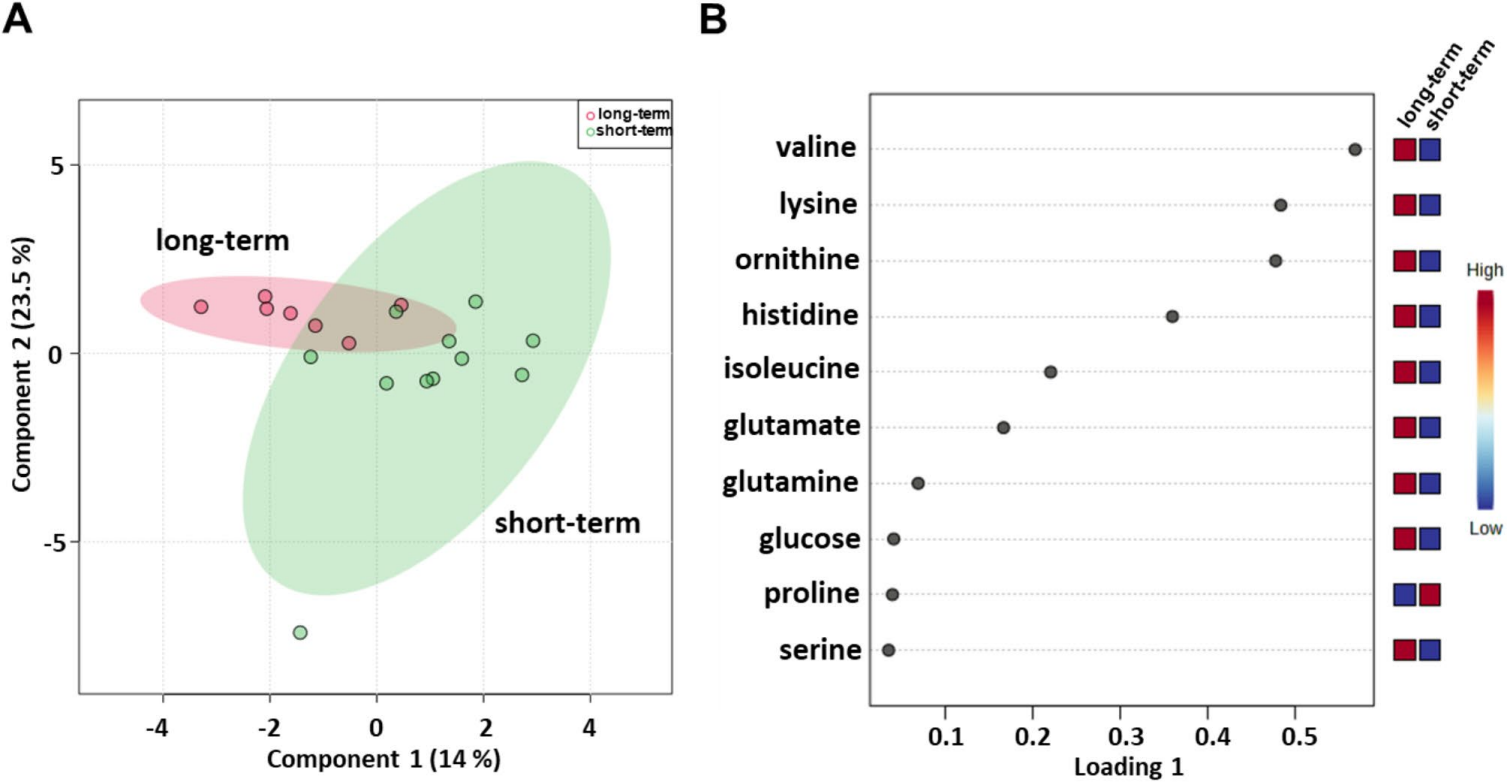
Score plot (**A**) and loading plot (**B**) related to metabolomic profiling of the sera of metastatic melanoma patients treated with nivolumab at first-line enrolled in training set, and subdivided accordingly to OS in good (long-term; OS ≥ 1 year) and poor (short-term; OS < 1 year) outcome

Next, we evaluated serum metabolomic profiles of baseline samples from 18 (7 long-term and 11 short-term OS) metastatic melanoma patients treated with nivolumab (Table 1). sPLS-DA (37.5% of the total variance) revealed that the long-term compared with short-term metabolomics profiles grouped into two different clusters (Fig. 5A) also for this cohort of patients. Lower levels of proline and higher levels of glucose, glutamate, glutamine, histidine, isoleucine, lysine, ornithine, serine and valine were detected in the long-term OS group (Fig. 5B).

Moreover, metabolite-set enrichment analysis revealed that the urea cycle; ammonia recycling; glucose‒alanine cycle; arginine and proline metabolism; the Warburg effect; valine, leucine and isoleucine degradation; lysine degradation; amino sugar metabolism; beta-alanine metabolism; methylhistidine metabolism; aspartate metabolism; nicotinate and nicotinamide metabolism; and propanoate metabolism were pathways that discriminated the serum metabolic profiles of the two groups (Fig. S7; Table S3).

**Fig. 6:**
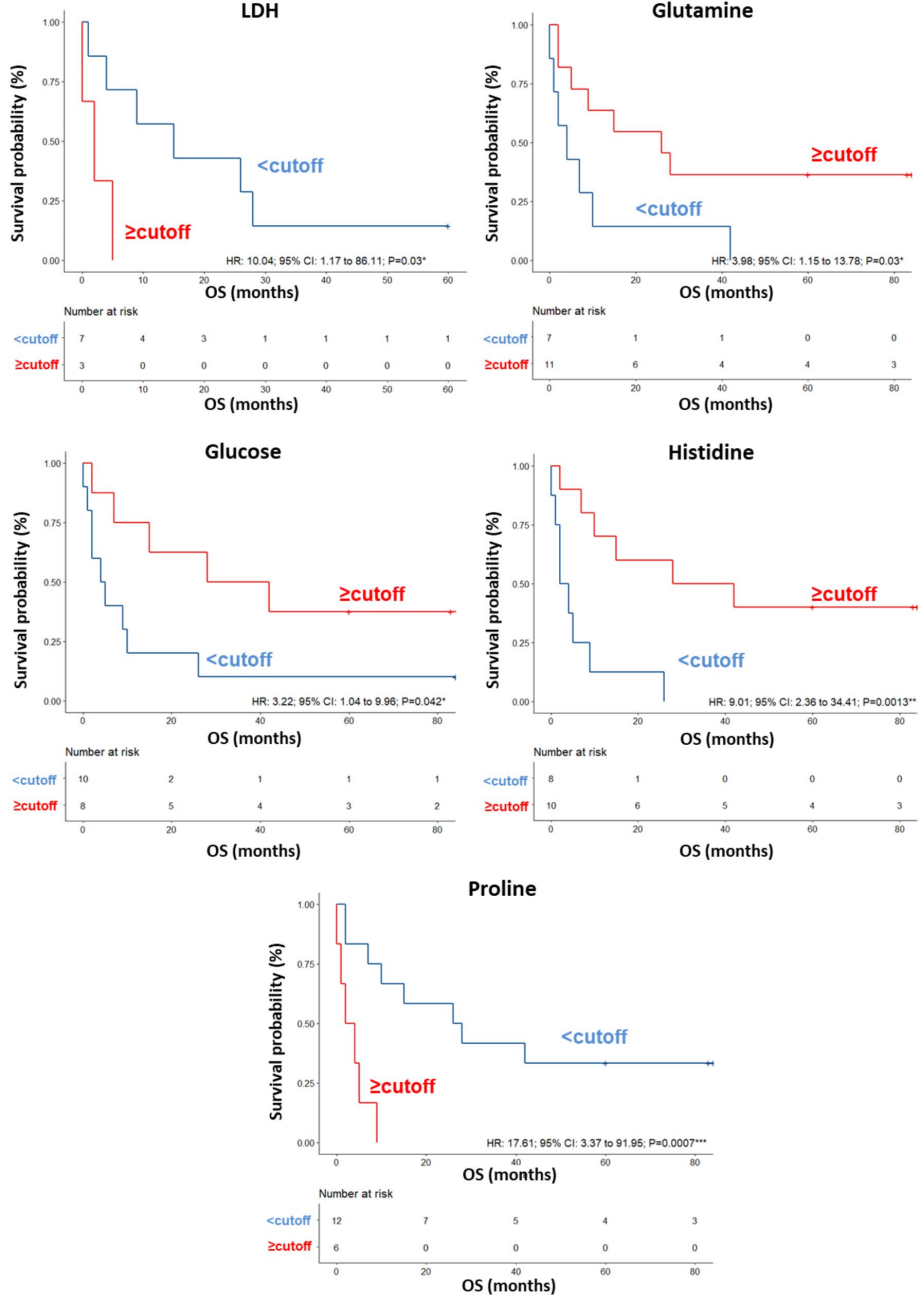
Kaplan–Meier curves of OS accordingly to LDH, glutamine, glucose, histidine and proline in the sera of metastatic melanoma patients treated with nivolumab at first-line enrolled in training set (log-rank p values are reported). *, ** and *** symbols indicate p values < 0.05, < 0.01 and < 0.0001, respectively

A validation set of additional 23 patients was also tested, confirming a clear separation in the score plot between 12 long-term OS and 11 short-term OS patients (Fig. S8A), and the same metabolites identified in the training set correlating with OS (Fig. S8B). ROC curve analysis revealed that the AUC values for the metabolites ranged between 0.695 and 0.857 (Fig. S9). In this group of patients, the univariate analysis demonstrated that LDH levels (HR: 10.04; 95% CI: 1.17–86.11; *P* = 0.03) were significantly associated with OS. No significant associations were found between OS and the other parameters reported in Table 6. Among the metabolites, higher (≥ cutoff) levels of glutamine (HR: 3.98; 95% CI: 1.15–13.78; *P* = 0.030), glucose (HR: 3.22; 95% CI: 1.04–9.96; *P* = 0.042) and histidine (HR: 9.01; 95% CI: 2.36–34.41; *P* = 0.0013) as well as lower (< cutoff) levels of proline (HR: 17.61; 95% CI: 3.37–91.95; *P* = 0.0007) predicted OS (Table 6; Fig. 6). In the multivariate analysis, histidine was the only parameter that significantly predicted OS (Table 6). In this cohort of patients, we did not find any association between response to nivolumab and the levels of the significant metabolites measured in pretreatment sera (Table 7).

**Table 6:** Univariate and multivariate analyses of baseline patient characteristics and metabolites for OS in patients treated with nivolumab at first line

	Univariate	Multivariate
	HR (95% CI) *P* value	HR (95% CI) *P* value
Patients characteristics		
Gender (F vs. M)	1.25(0.30–5.28) *P* = 0.76	
Age, years (> 50 vs. < = 50)	1.25(0.17–8.99) *P* = 0.82	
BRAF status (mut vs. wt)	1.24(0.26–5.99) *P* = 0.78	
LDH, IU/L (≥ 480 vs. < 480)	10.04(1.17–86.11) ***P*** = 0.03*	38.84(0.14–73.82) *P* = 0.95
Brain metastates (yes vs. no)	7.71(0.65–92.02) *P* = 0.10	
Metabolites (nps)		
Glutamine level (<-0.0945 vs≥--0.0945)	3.98(1.15–13.78) ***P*** = 0.030*	1.14(0.26–10.14) *P* = 0.95
Glutamate level (< 0.422 vs≥-0.422)	2.48(0.82–7.44) *P* = 0.11	
Glucose level (< 0.363 vs≥-0.363)	3.22(1.04–9.96) ***P*** = 0.042*	1.39(0.24–10.14) *P* = 0.99
Isoleucine level (< 0.861vs ≥-0.861)	1.69(0.57–5.06) *P* = 0.34	
Histidine level (< 0.515vs ≥-0.515)	9.01(2.36–34.41) ***P*** = 0.0013**	4.64(2.79–84.24) ***P*** = 0.031*
Lysine level (< 0.185 vs. ≥--0.185)	2.65(0.81–8.68) *P* = 0.11	
Ornithine level (< 0.813 vs. ≥-0.813)	1.98(0.63–6.15) *P* = 0.24	
Proline level (≥-0.61 vs. < 0.61)	17.61(3.37–91.95) ***P*** = 0.0007***	2.35(0.85-135.08) *P* = 0.99
Serine level (< 0.141 vs≥--0.141)	2.03(0.61–6.71) *P* = 0.25	
Valine level (< 0.686 vs≥-0.686)	2.36(0.78–7.06) *P* = 0.13	

HR: hazard ratio; CI: confidence interval; nps: normalized values of the proton signals. M: male; F: female; mut: mutant; wt: wild type; LDH: lactate dehydrogenase; IU/L: International Units per Liter; Significant p-values < 0.05 are reported in bold

**Table 7:** Response to treatment related to the levels of the significant metabolites for patients treated with nivolumab at first line

	CR	PR	SD	PD	DCR(CR + PR + SD)	*p*-value; OR(95%CI)§
Patient number	1	5	3	9	9	
Glutamine level (<-0.0945)	1	1	1	4	3	0.63
Glutamine level (≥-0.0945)		4	2	5	6	
Glutamate level (< 0.422)		2	2	9	4	0.068
Glutamate level (≥ 0.422)	1	3	1		5	
Glucose level (< 0.363)		2	2	6	4	0.34
Glucose level (≥ 0.363)	1	3	1	3	5	
Isoleucine level (< 0.861)	1	1	3	7	5	0.31
Isoleucine level (≥ 0.861)		4		2	4	
Histidine level (< 0.515)		2	1	5	3	0.34
Histidine level (≥ 0.515)	1	3	2	4	6	
Lysine level (<-0.185)		1	2	5	3	0.34
Lysine level (≥-0.185)	1	4	1	4	6	
Ornithine level (< 0.813)		4	2	8	6	0.26
Ornithine level (≥ 0.813)	1	1	1	1	3	
Proline level (< 0.61)	1	4	2	5	7	0.32
Proline level (≥ 0.61)		1	1	4	2	
Serine level (<-0.141)		2	1	4	3	0.63
Serine level (≥-0.141)	1	3	2	5	6	
Valine level (< 0.686)		2	3	7	5	0.32
Valine level (≥ 0.686)	1	3		2	4	

CR: complete response; PR: partial response; SD: stable response; PD: progression disease; DCR: disease control rate; ^§^Association between the response and the metabolites levels was evaluated by chi square test. When the association is statistically significant (p-value < 0.05), we reported also odds ratio (OR) and 95% confidence interval (CI)

### Identification of baseline serum metabolites that can predict the outcome of patients treated with ipilimumab plus nivolumab combination in the first-line setting

**Fig. 7:**
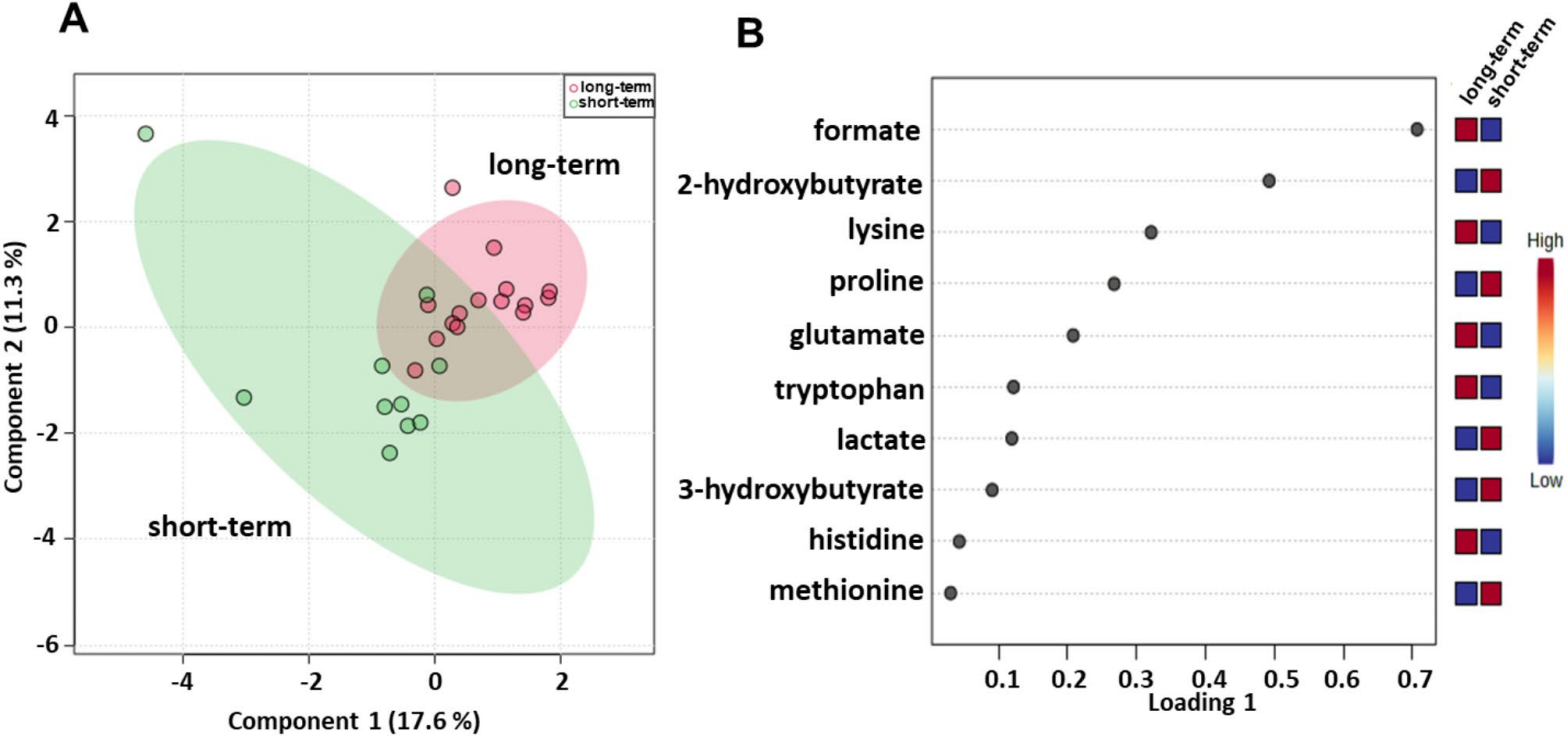
Score plot (**A**) and loading plot (**B**) related to metabolomic profiling of the sera of metastatic melanoma patients treated with ipilimumab plus nivolumab at first-line enrolled in training set, and subdivided accordingly to OS in good (long-term; OS ≥ 1 year) and poor (short-term; OS < 1 year) outcome

We subsequently evaluated the serum metabolomic profiles of baseline samples from 25 (15 long-term and 10 the short-term OS) metastatic melanoma patients treated with ipilimumab plus nivolumab in the first-line setting (Table 1). sPLS-DA (28.9% of the total variance) revealed that the long-term compared with short-term metabolomics profiles grouped into two different clusters (Fig. 7A) and the loading plot revealed lower levels of 2-hydroxybutyrate, 3-hydroxybutyrate, lactate, methionine and proline and higher levels of formate, glutamate, histidine, lysine and tryptophan in the long-term OS group (Fig. 7B). Metabolite set enrichment analysis revealed that tryptophan metabolism, folate metabolism, lysine degradation, ammonia recycling, methylhistidine metabolism, and beta-alanine metabolism were pathways that discriminated the serum metabolic profiles of the two groups (Fig. S10; Table S4).

A validation set of 18 patients was also tested for this treatment group, confirming a clear separation in the score plot between the profiling of 10 long-term and 8 short-term OS patients (Fig. S11A) with the same metabolites identified in the training set correlating with OS (Fig. S11B).

**Fig. 8:**
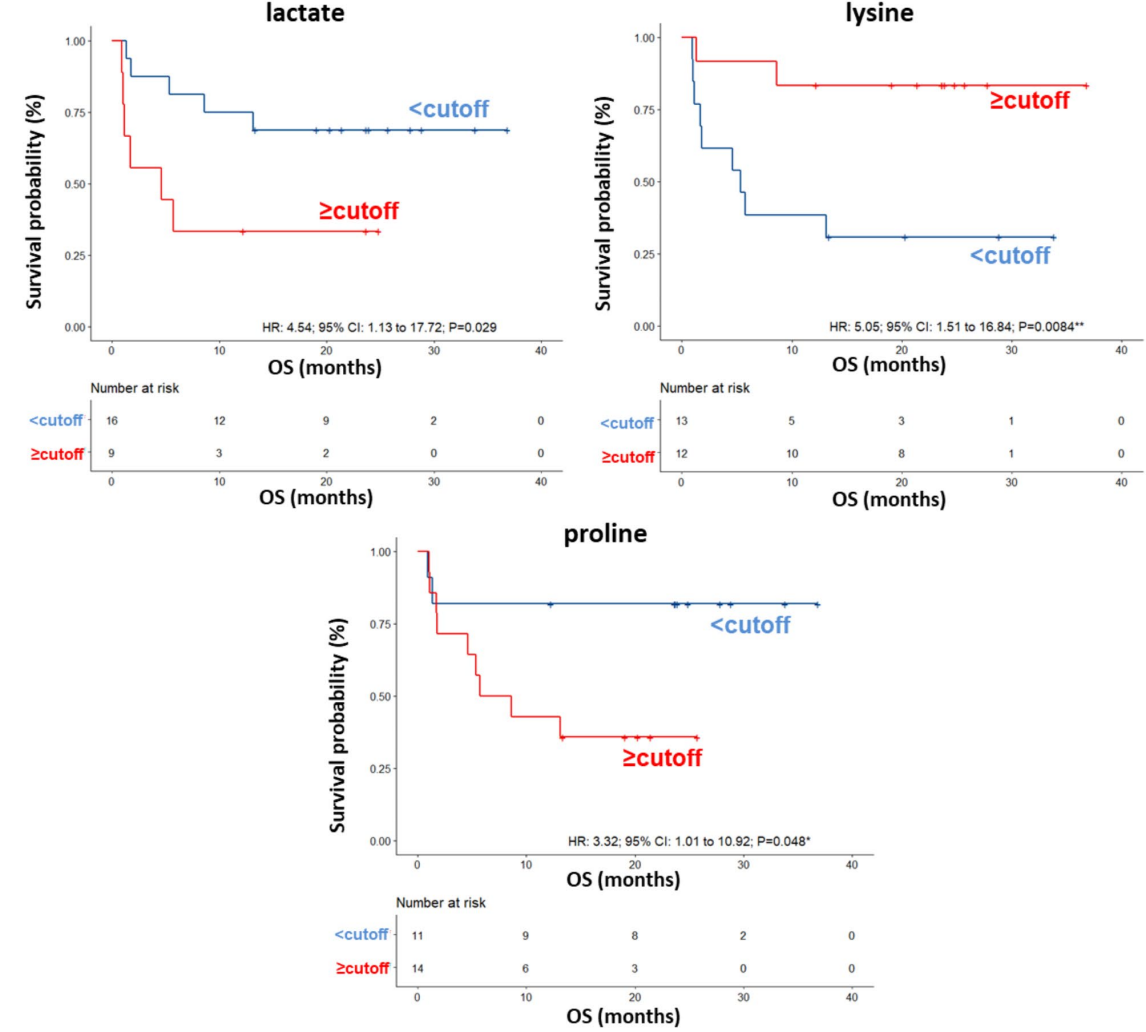
Kaplan–Meier curves of OS accordingly to lactate, lysine and proline in the sera of metastatic melanoma patients treated with ipilimumab plus nivolumab at first-line enrolled in training set (log-rank p values are reported). * and ** symbols indicate p values < 0.05 and < 0.01, respectively

ROC curve analysis revealed that the AUC values for the metabolites ranged between 0.573 and 0.76 (Fig. S12) and univariate analysis revealed no significant associations between OS of this group of patients and the parameters reported in Table 8. Among the metabolites, lower (< cutoff) levels of lactate (HR: 4.54; 95% CI: 1.16–17.72; *P* = 0.029) and proline (HR: 3.32; 95% CI: 1.01–10.92; *P* = 0.048) as well as higher (≥ cutoff) levels of lysine (HR: 5.05; 95% CI: 1.51–16.84; *P* = 0.0084) were correlated with a more favorable OS (Table 8; Fig. 8). In the multivariate analysis, lower levels of lactate were the only parameter that significantly predicted favorable OS (Table 8). Accordingly, also the DCR of ipilimumab plus nivolumab was associated with low levels of lactate (OR: 0.13; 95% CI: 0.020–0.86; *P* = 0.025) (Table 9).

**Table 8:** Univariate and multivariate analyses of baseline patient characteristics and metabolites for OS in patients treated with ipilimumab plus nivolumab at first line

	Univariate	Multivariate
	HR (95% CI) *P* value	HR (95% CI) *P* value
**Patients characteristics**		
**Gender** (M vs. F)	1.95(0.59–6.47) *P* = 0.27	-
**Age**,** years** ( < = 50 vs. > 50)	2.66(0.72–9.79) *P* = 0.14	-
**BRAF status** (mut vs. wt)	1.10(0.31–3.86) *P* = 0.88	-
**LDH**,** IU/L** (≥ 480 vs. < 480)	0.88(0.23–3.34) *P* = 0.86	-
**Brain metastates**(no vs. yes)	-	-
**Metabolites(nps)**		
**2-hydroxybutyrate level** (≥-0.094vs <-0.094)	2.61(0.80–8.58) *P* = 0.11	-
**3-hydroxybutyrate level** (≥-0.26vs <-0.26)	2.35(0.72–7.68) *P* = 0.16	-
**Formate level** (<-0.41 vs. ≥-0.41)	2.50(0.74–8.38) *P* = 0.14	-
**Glutamate level** (< 1.1 vs. ≥ 1.1)	3.40(0.99–11.62) *P* = 0.051	-
**Histidine level** (<-0.44 vs. ≥-0.44)	1.22(0.29–5.15) *P* = 0.78	-
**Lactate level** (≥ 0.44vs < 0.44)	4.54(1.16–17.72) ***P*** = 0.029*	7.94(1.93–32.60) ***P*** = 0.0041**
**Lysine level** (< 0.41 vs. ≥ 0.41)	5.05(1.51–16.84) ***P*** = 0.0084**	1.39(0.86–11.90) *P* = 0.072
**Methionine level** (≥-0.11vs <-0.11)	1.38(0.42–4.51) *P* = 0.60	-
**Proline level** (≥ 0.039vs < 0.039)	3.32(1.01–10.92) ***P*** = 0.048*	5.72(0.89–29.89) *P* = 0.069
**Tryptophan level** (<-0.083 vs. ≥-0.083)	2.46(0.65–9.28) *P* = 0.18	-

**Table 9:** Response to treatment related to the levels of the significant metabolites for patients treated with ipilimumab plus nivolumab at first line

	CR	PR	SD	PD	DCR(CR + PR + SD)	*p*-value; OR(95%CI)^§^
**Patient number**	3	6	4	12	13	
**2-hydroxybutyrate** (<-0.094)	2	3	3	3	8	0.066
**2-hydroxybutyrate** (≥-0.094)	1	3	1	9	5	
**3-hydroxybutyrate** (<-0.26)	2	3	1	5	6	0.82
**3-hydroxybutyrate** (≥-0.26)	1	3	3	7	7	
**Formate** (<-0.41)	1	1	1	9	3	0.051
**Formate** (≥-0.41)	2	5	3	3	10	
**Glutamate** (< 1.1)	2	1	4	10	7	0.11
**Glutamate** (≥ 1.1)	1	5		2	6	
**Histidine** (<-0.44)	2	4	4	10	10	0.69
**Histidine** (≥-0.44)	1	2		2	3	
**Lactate** (< 0.44)	2	5	4	5	11	0.025*;0.13(0.020–0.86)
**Lactate** (≥ 0.44)	1	1		7	2	
**Lysine** (< 0.41)	1	2	2	8	5	0.38
**Lysine** (≥ 0.41)	2	4	2	4	8	
**Methionine** (<-0.11)	2	3	3	5	8	0.32
**Methionine** (≥-0.11)	1	3	1	7	5	
**Proline** (< 0.039)	3	4	1	3	8	0.066
**Proline** (≥ 0.039)		2	3	9	5	
**Tryptophan** (<-0.083)	2	1		5	3	0.32
**Tryptophan** (≥-0.083)	1	5	4	7	10	

CR: complete response; PR: partial response; SD: stable response; PD: progression disease; DCR: disease control rate; ^§^Association between the response and the metabolites levels was evaluated by chi square test. When the association is statistically significant (p-value < 0.05), we reported also odds ratio (OR) and 95% confidence interval (CI)

### Comparison of the results obtained in the different treatment subgroups highlighted critical common metabolites that predict outcome of ICIs therapy

To identify the common significant metabolites in the three different treatment subgroups, we compared the results using a Venn diagram. As shown in Fig. S13, higher pretreatment levels of histidine were commonly found in long-term OS subgroups of patients treated with ipilimumab, nivolumab or ipilimumab plus nivolumab; higher pretreatment basal levels of isoleucine, glucose and valine were detected in long-term OS subgroups of patients treated with ipilimumab or nivolumab; and higher pretreatment basal levels of glutamate and lysine and lower proline were detected in long-term OS subgroups of patients treated with nivolumab or ipilimumab plus nivolumab. Interestingly, all these metabolites were involved in those pathways enriched with at least three metabolites (Tables S2, S3, and S4): ammonia recycling, the Warburg effect, tryptophan metabolism, the urea cycle, and valine, leucine, and isoleucine degradation pathways.

In any case, interestingly, if we considered only those metabolites that predict OS after univariate analysis, lower lactate levels were associated with favorable OS in the total population and in the ipilimumab plus nivolumab combination cohort, being in this latter cohort also an independent predictor of OS after multivariate analysis. Moreover, lower proline levels were associated with favorable OS in both the nivolumab and ipilimumab plus nivolumab cohorts, while higher histidine levels were associated with favorable OS in both the ipilimumab and nivolumab cohorts (Fig. S14).

## Discussion

A significant number of patients with metastatic melanoma fail to benefit from ICI treatments and frequently develop resistance. Consequently, identifying circulating blood biomarkers that can improve patient prognosis and predict the outcomes of patients receiving ICI therapy is essential. Metabolic reprogramming is closely linked to cancer initiation and progression and emerged as a critical hallmark of cancer [23]. Indeed metabolomics approaches in recent years have been considered useful tools for identifying metabolites capable of distinguishing different stages of tumor disease, predicting prognosis or stratifying patients responsive or non-responsive to anti-tumor treatments [24]. In the present study, using an untargeted NMR-based metabolomics approach, we identified pretreatment serum metabolites that are able to predict OS in metastatic melanoma patients receiving first-line therapy with nivolumab, ipilimumab or their combination.

A previous NMR-based targeted metabolomics study reported significant differences in baseline serum metabolite profiles of 26 advanced-stage (III and IV) melanoma patients compared to 46 healthy age- and sex-matched controls [19]. Our group obtained similar data using an untargeted NMR-based approach to analyze serum metabolomics in patients with different stages of melanoma and matched healthy controls (unpublished observations).

Recently, using a mass spectrometry (MS)-based targeted metabolomics platform, researchers demonstrated that in a heterogeneous cohort of 36 cutaneous, mucosal or uveal melanoma patients, there were significant differences in the levels of circulating metabolites depending on the melanoma subtype and outcomes after ICI treatment [14]. Interestingly, Triozzi et al. (2022), by evaluating the bioenergetics of circulating immune cells as well as the MS-measured serum metabolomic profile in samples collected at baseline from 40 melanoma patients treated with anti-PD1 therapy, suggested that a glycolytic signature is associated with checkpoint inhibitor responders [25].

However, the present study, to the authors’ knowledge, is the first to explore serum metabolomics signatures associated with OS in metastatic melanoma patients treated with different immune checkpoint inhibitor therapies. We identified potential metabolic biomarkers that are detectable inpatient serum before treatment and can discriminate short-term versus long-term survival after ICI therapy.

Some of these metabolites were significantly correlated with OS after univariate analysis: ii) lactate, tryptophan and valine in all patients subjected to ICIs immunotherapy; ii) alanine, asparagine, glutathione, histidine, isoleucine and phenylalanine in patients treated with ipilimumab; iii) glucose, glutamine, histidine and proline in patients treated with nivolumab; iv) lactate, lysine and proline in patients treated with ipilimumab plus nivolumab. Interestingly, lower lactate levels were associated with favorable OS in the total population and in the ipilimumab plus nivolumab combination cohort, being in this latter cohort also an independent predictor of OS after multivariate analysis. Moreover, lower proline levels were associated with favorable OS in both the nivolumab and ipilimumab plus nivolumab cohorts, while higher histidine levels were associated with favorable OS in both the ipilimumab and nivolumab cohorts, being in this latter cohort also an independent predictor of OS after multivariate analysis. Lower levels of glutathione and phenylalanine and higher levels of tryptophan were also independent predictors of OS after multivariate analysis in ipilimumab cohort and in the total population, respectively. Notably, those metabolites that were significantly associated with OS after a multivariate analysis also correlated with response to ICI treatment. In details, tryptophan correlated with ICI-induced DCR in all the patients’ population, and histidine and lactate in patients treated with ipilimumab and with ipilimumab plus nivolumab, respectively.

Importantly, the metabolites associated with patient outcomes in our study have already been correlated as dysregulated in melanoma. Indeed, lower levels of asparagine, glucose, glutamine, histidine, isoleucine and lysine and higher levels of alanine, glutathione, lactate, phenylalanine and proline have been found in the sera of stage III and IV melanoma patients than in the sera of healthy controls [19].

Knowledge of the role of these molecules in human metabolism and how they are associated with patient outcomes and/or responses to treatment is fundamental to understanding how their detection can elucidate the metabolic abnormalities within the tumor microenvironment that may hinder the ability to trigger an effective antitumor response.

The correlation between glucose and lactate levels and melanoma patient outcomes in our study confirms that melanoma cells increase glucose metabolism, repress oxidative phosphorylation, and are capable of metabolizing glucose to lactose even in the presence of oxygen in the process of aerobic glycolysis, as already reported in the literature [26].

Glucose metabolism is regulated by oncogenic BRAF through hypoxia inducible factor 1 alpha (HIF-1 alpha), avian myelocytomatosis viral oncogene homolog (c-Myc), and microphthalmia-associated transcription factor (MITF), and BRAF-mutated melanomas exhibit increased glycolysis [27].

Importantly, immune cell reprogramming mediated by lactate is known to increase immunosuppression in the tumor microenvironment (TME) and represents a key factor in regulating tumor development, metastasis, and the effectiveness of ICI-based immunotherapies. Indeed, lactate participates in regulating PD-1 and programmed death-Ligand (PD-L)1 expression, thereby exerting immunosuppressive effects and affecting the therapeutic effect of ICIs. Therefore, anti-lactate combined immunotherapy appears to be a promising treatment modality [28]. Recently, high serum levels of lactate dehydrogenase (LDH), an important enzyme of the anaerobic metabolic pathway that converts pyruvate, the final product of glycolysis, to lactate, were shown to be associated with a poorer prognosis in metastatic melanoma patients treated with immunotherapy, regardless of the presence of brain metastases [11]. On the other hand, lactate should be considered the primary carbohydrate fuel in responder patients because its reverse conversion to pyruvate can produce nicotinamide adenine dinucleotide (NADH), which can be transported to mitochondria to support electron transport, ATP production and the reprogramming of fatty acid and amino acid metabolism [25]. Interestingly, the increase in both glucose uptake rate and lactate production is associated with the Warburg effect, which has been reported to limit the success of immunotherapeutic approaches by blocking the anti-tumor function of NK and T cells and promoting the activity of immunosuppressive cell populations such as MDSCs and Tregs [29]. A correlation between high glycolytic activity and poor response to anti-PD-1 therapy has been reported in both renal carcinoma and stage IV melanoma patients [30, 31]. Notably, it has been demonstrated that targeting the Warburg effect by LDH suppression enhances the efficacy of anti-PD1 therapy in murine melanoma models [32].

An interesting point of discussion concerns the fact that most amino acids, including isoleucine, valine and phenylalanine, as well as tryptophan, are transported by L-type amino acid transporter 1 (LAT1). The potential prognostic significance of LAT1 has been explored in melanoma patients, and the results indicated that high LAT1 expression is correlated with CD98, cell proliferation (Ki-67), and microvessel density (CD34) and with OS and disease-free survival. In particular, a multivariate analysis confirmed that LAT1 was an independent prognostic factor for predicting a poor prognosis [33]. Hence, an evaluation of LAT1 together with the levels of branched chain amino acid (BCAA), phenylalanine and tryptophan could be used as a predictor of OS in melanoma patients.

Importantly, tryptophan is an essential amino acid of significant interest in cancer research and immunotherapy for several reasons: the high expression of tryptophan-degrading enzymes, such as Trp 2,3-dioxygenase (TDO) and indoleamine 2,3-dioxygenase (IDO), in cancer cells; the production of the immunomodulatory kynurenine metabolite resulting from tryptophan degradation; and the regulation of tryptophan levels by tumors to suppress immune defenses, thereby facilitating immune escape [34]. Altered tryptophan metabolism has been studied in melanoma patients, and the results revealed that low levels of tryptophan in the serum are correlated with increased levels of kynurenine [35]. The blockade of immunosuppressive tryptophan degradation mediated by IDO and TDO represents great promise for sensitizing cancer patients to immune checkpoint blockade. However, while early clinical studies of combined PD1 and IDO1 inhibition in metastatic melanoma have shown promising results [36], another randomized clinical study has failed to demonstrate a more significant clinical benefit from this combination than PD1 inhibition alone [37]. For this reason, some researchers are focusing on an alternative pathway of tryptophan metabolism, known as the serotonergic pathway. In this pathway, tryptophan is metabolized by Trp hydroxylase (TPH) 1 and TPH2 to produce serotonin (5HT) and melatonin. Melanoma cells can express TPH enzymes and produce serotonin and melatonin [38]. In a prospective clinical trial of pembrolizumab in metastatic melanoma patients (NCT03089606), increased tryptophan metabolism was confirmed to be a poor predictor of pembrolizumab response and an adverse prognostic factor. Additionally, the activation of the serotoninergic pathway was found to be more significant than the kynurenine pathway in predicting patient outcomes [39].

Overall our data strongly suggest that the higher levels of asparagine, isoleucine, glutamine, lysine, tryptophan and valine and lower levels of alanine, glutathione, phenylalanine and proline resulted to be associated with favorable OS, are closely related to each other, and involved in cancer-associated metabolic reprogramming. In support of this, previous studies have demonstrated similar trends. For example, higher tryptophan concentrations have been shown to be significantly correlated with better OS in non-small cell lung cancer (NSCLC) patients prior to treatment with PD-1 inhibitors [40]. Additionally, the levels of glutamate/glutamine, phenylalanine, n-acetyl-d-tryptophan and valine have been shown to be significantly increased in NSCLC patients after treatment with PD-1/PD-L1 inhibitors [41]. Taken together, these published findings align with the results of our study, which identified elevated levels of these metabolites in long-term OS patients, further substantiating the relationship between metabolic profiles and treatment outcomes.

An additional point of discussion concerns the results obtained from the comparison between the significant metabolites identified in all the subgroups of patients treated with different ICI strategies in the first-line setting and the metabolic pathways in which these metabolites are involved. Notably, histidine was also the only metabolite whose higher pretreatment levels were commonly found in long-term OS subgroups of all the three cohorts of patients (ipilimumab, nivolumab or ipilimumab plus nivolumab treatment). Histidine is an essential amino acid associated with increased inflammation and oxidative stress [42]. Lower levels of histidine have been attributed to higher histidine decarboxylase activity, resulting in the accelerated decarboxylation of histidine to histamine, a mediator involved in inflammatory and immune responses associated with cancer initiation and progression [43]. Taylor et al. (2020) compared the metabolic profiles of primary and metastatic melanoma tissues with those of matched extratumoral microenvironment (EM) tissues. The authors demonstrated that the histidine metabolic pathway distinguished metastatic melanoma from EM but not primary melanoma from EM, suggesting that this pathway may be important for melanoma progression rather than initiation [44]. However, higher histidine levels in early on-treatment serum from advanced NSCLC patients receiving anti-PD1 therapy are associated with favorable OS and PFS; thus, histidine has been proposed as a potential predictive biomarker of the response to PD-1 blockade therapy in advanced NSCLC patients [45]. Besides, histidine plays a key role in ammonia recycling metabolism, helping to regulate ammonia levels and supporting the increased nitrogen metabolism commonly observed in tumors. Interestingly, recently it has been identified a novel form of T-cell death, ammonia-induced cell death, which may have significant implication for immunotherapy efficacy. Ammonia produced from glutamine and asparagine catabolism, accumulates in effector CD8 + T cells and triggers their apoptosis. As a result, the efficacy of immunotherapy is reduced due to the low survival rate of effector CD8 + T cells after they have exerted their antitumor effects [46, 47].

## Conclusions

In this study, we identified metabolomic signatures in pretreatment sera that can distinguish patients with metastatic melanoma who have good outcomes after ICI treatment from those who do not. Hence, our results contribute to the understanding of response and resistance mechanisms to ICI, suggesting a novel approach to support further personalized treatment strategies in the management of melanoma patients. Indeed, an ideal biomarker would have both predictive utility and the ability to be targeted therapeutically.

Finally we are aware that the promising results obtained in this retrospective single center study have several limitations and need to be validated in a larger cohort of patients from a multicenter prospective study.

## Electronic supplementary material

Below is the link to the electronic supplementary material.

Supplementary Material 1

Supplementary Material 2

## Data Availability

The datasets presented in this study can be found in online repositories. The names of the repository/repositories and accession number(s) can be found below: 10.5281/zenodo.5141301.

## Abbreviations

ATP: Adenosine triphosphate
AUC: Area under the curve
BCAA: Branched chain amino acid
CI: Confidence intervals
c-Myc: Avian myelocytomatosis viral oncogene homolog
CPMG: Carr-Purcell-Meiboom-Gill
CR: Complete response
CTLA-4: Cytotoxic T-Lymphocyte Antigen 4
DCR: Disease control rate
EM: Extratumoral microenvironment
FDA: Food and Drug Administration
FoxP3: Forkhead box P3
HIF-1 alpha: Hypoxia inducible factor 1 alpha
HMDB: Human Metabolome Database
HR: Hazard ratio
ICI: Immune checkpoint inhibitor
IDO: Indoleamine 2,3-dioxygenase
K-M: Kaplan-Meier
LAT1: L-type amino acid transporter 1
LDH: Lactate dehydrogenase
MITF: Microphtalmia-associated transcription factor
MM: Metastatic melanoma
MS: Mass spectrometry
NADH: Nicotinamide adenin dinucleotide
NLR: Neutrophil/lymphocyte ratio
NMR: Nuclear Magnetic Resonance
NSCLC: Non-small cell lung cancer
OR: Odds ratio
ORR: Objective response rate
OS: Overall survival
PBS: Phosphate-buffered saline
PD: Progressive disease
PD-1: Programmed cell death protein 1
PD-L1: Programmed Death-Ligand 1
PFS: Progression-free survival
PR: Partial response
P: P-value
RECIST: Response Evaluation Criteria In Solid Tumors
ROC: Receiver-operating characteristic
sPLS-DA: Sparse partial least squares discriminant analysis
SD: Stable disease
SVM: Support vector machines
TCA: Tricarboxylic acid
TDO: Trp 2,3-dioxygenase
TGFβ: Transforming Growth Factor Beta
TME: Tumor microenvironment
TPH: Tryptophan hydroxylase
TSP: Sodium 3-trimethylsilyl [2,2,3,3-2H4] propionate
VIP: Variable importance plot

## References

[1] Luther C, Swami U, Zhang J, Milhem M, Zakharia Y. Advanced stage melanoma therapies: detailing the present and exploring the future. Crit Rev Oncol Hematol. 2019;133:99–111. 30661664 10.1016/j.critrevonc.2018.11.002

[2] Hodi FS, O’Day SJ, McDermott DF, Weber RW, Sosman JA, Haanen JB, et al. Improved survival with ipilimumab in patients with metastatic melanoma. N Engl J Med. 2010;363(8):711–23. 20525992 10.1056/NEJMoa1003466 PMC3549297

[3] Long GV, Carlino MS, McNeil C, Ribas A, Gaudy-Marqueste C, Schachter J, et al. Pembrolizumab versus ipilimumab for advanced melanoma: 10-year follow-up of the phase III KEYNOTE-006 study. Ann Oncol. 2024;35(12):1191–9. 39306585 10.1016/j.annonc.2024.08.2330

[4] Wolchok JD, Chiarion-Sileni V, Rutkowski P, Cowey CL, Schadendorf D, Wagstaff J, et al. Final, 10-Year outcomes with nivolumab plus ipilimumab in advanced melanoma. N Engl J Med. 2025;392(1):11–22. 39282897 10.1056/NEJMoa2407417 PMC12080919

[5] Carlino MS, Larkin J, Long GV. Immune checkpoint inhibitors in melanoma. Lancet. 2021;398(10304):1002–14. 34509219 10.1016/S0140-6736(21)01206-X

[6] Regan MM, Mantia CM, Werner L, Tarhini AA, Larkin J, Stephen Hodi F et al. Treatment-free survival over extended follow-up of patients with advanced melanoma treated with immune checkpoint inhibitors in checkmate 067. J Immunother Cancer. 2021;9(11). 10.1136/jitc-2021-003743 PMC8606772 34799400

[7] Bai R, Lv Z, Xu D, Cui J. Predictive biomarkers for cancer immunotherapy with immune checkpoint inhibitors. Biomark Res. 2020;8:34. 32864131 10.1186/s40364-020-00209-0 PMC7450548

[8] Martens A, Wistuba-Hamprecht K, Geukes Foppen M, Yuan J, Postow MA, Wong P, et al. Baseline peripheral blood biomarkers associated with clinical outcome of advanced melanoma patients treated with ipilimumab. Clin Cancer Res. 2016;22(12):2908–18. 26787752 10.1158/1078-0432.CCR-15-2412 PMC5770142

[9] Bartlett EK, Flynn JR, Panageas KS, Ferraro RA, Sta Cruz JM, Postow MA, et al. High neutrophil-to-lymphocyte ratio (NLR) is associated with treatment failure and death in patients who have melanoma treated with PD-1 inhibitor monotherapy. Cancer. 2020;126(1):76–85. 31584709 10.1002/cncr.32506 PMC6906249

[10] Weide B, Martens A, Hassel JC, Berking C, Postow MA, Bisschop K, et al. Baseline biomarkers for outcome of melanoma patients treated with pembrolizumab. Clin Cancer Res. 2016;22:5487–96. 27185375 10.1158/1078-0432.CCR-16-0127 PMC5572569

[11] Mallardo D, Fordellone M, White A, Ottaviano M, Sparano F, Bailey M, et al. CD39 and LDHA affects the prognostic role of NLR in metastatic melanoma patients treated with immunotherapy. J Transl Med. 2023;21(1):610. 37684649 10.1186/s12967-023-04419-6 PMC10492378

[12] Hu T, Liu CH, Lei M, Zeng Q, Li L, Tang H, et al. Metabolic regulation of the immune system in health and diseases: mechanisms and interventions. Signal Transduct Target Ther. 2024;9(1):268. 39379377 10.1038/s41392-024-01954-6 PMC11461632

[13] Aderinto N, Abdulbasit MO, Tangmi ADE, Okesanya JO, Mubarak JM. Unveiling the growing significance of metabolism in modulating immune cell function: exploring mechanisms and implications; a review. Ann Med Surg (Lond). 2023;85(11):5511–22. 37915697 10.1097/MS9.0000000000001308 PMC10617839

[14] Vilbert M, Koch EC, Rose AAN, Laister RC, Gray D, Sotov V et al. Analysis of the Circulating metabolome of patients with cutaneous, mucosal and uveal melanoma reveals distinct metabolic profiles with implications for response to immunotherapy. Cancers (Basel). 2023;15(14). 10.3390/cancers15143708 PMC10378038 37509369

[15] Wang W, Zhen S, Ping Y, Wang L, Zhang Y. Metabolomic biomarkers in liquid biopsy: accurate cancer diagnosis and prognosis monitoring. Front Oncol. 2024;14:1331215. 38384814 10.3389/fonc.2024.1331215 PMC10879439

[16] Kaushik AK, DeBerardinis RJ. Applications of metabolomics to study cancer metabolism. Biochim Biophys Acta Rev Cancer. 2018;1870(1):2–14. 29702206 10.1016/j.bbcan.2018.04.009 PMC6193562

[17] Costantini S, Di Gennaro E, Capone F, De Stefano A, Nasti G, Vitagliano C, et al. Plasma metabolomics, lipidomics and cytokinomics profiling predict disease recurrence in metastatic colorectal cancer patients undergoing liver resection. Front Oncol. 2022;12:1110104. 36713567 10.3389/fonc.2022.1110104 PMC9875807

[18] Markley JL, Bruschweiler R, Edison AS, Eghbalnia HR, Powers R, Raftery D, et al. The future of NMR-based metabolomics. Curr Opin Biotechnol. 2017;43:34–40. 27580257 10.1016/j.copbio.2016.08.001 PMC5305426

[19] Bayci AWL, Baker DA, Somerset AE, Turkoglu O, Hothem Z, Callahan RE, et al. Metabolomic identification of diagnostic serum-based biomarkers for advanced stage melanoma. Metabolomics. 2018;14(8):105. 30830422 10.1007/s11306-018-1398-9

[20] Costantini S, Madonna G, Di Gennaro E, Capone F, Bagnara P, Capone M et al. New insights into the identification of metabolites and cytokines predictive of outcome for patients with severe SARS-CoV-2 infection showed similarity with cancer. Int J Mol Sci. 2023;24(5). 10.3390/ijms24054922 PMC10003544 36902351

[21] Pang Z, Chong J, Zhou G, de Lima Morais DA, Chang L, Barrette M, et al. MetaboAnalyst 5.0: narrowing the gap between Raw spectra and functional insights. Nucleic Acids Res. 2021;49(W1):W388–96. 34019663 10.1093/nar/gkab382 PMC8265181

[22] Wishart DS, Jewison T, Guo AC, Wilson M, Knox C, Liu Y, et al. HMDB 3.0–The human metabolome database in 2013. Nucleic Acids Res. 2013;41(Database issue):D801–7. 23161693 10.1093/nar/gks1065 PMC3531200

[23] Pavlova NN, Zhu J, Thompson CB. The hallmarks of cancer metabolism: still emerging. Cell Metab. 2022;34(3):355–77. 35123658 10.1016/j.cmet.2022.01.007 PMC8891094

[24] Chang CH, Qiu J, O’Sullivan D, Buck MD, Noguchi T, Curtis JD, et al. Metabolic competition in the tumor microenvironment is a driver of cancer progression. Cell. 2015;162(6):1229–41. 26321679 10.1016/j.cell.2015.08.016 PMC4864363

[25] Triozzi PL, Stirling ER, Song Q, Westwood B, Kooshki M, Forbes ME, et al. Circulating immune bioenergetic, metabolic, and genetic signatures predict melanoma patients’ response to Anti-PD-1 immune checkpoint Blockade. Clin Cancer Res. 2022;28(6):1192–202. 35284940 10.1158/1078-0432.CCR-21-3114 PMC9179080

[26] Fischer GM, Vashisht Gopal YN, McQuade JL, Peng W, DeBerardinis RJ, Davies MA. Metabolic strategies of melanoma cells: mechanisms, interactions with the tumor microenvironment, and therapeutic implications. Pigment Cell Melanoma Res. 2018;31(1):11–30. 29049843 10.1111/pcmr.12661 PMC5742019

[27] Warrier G, Lanceta L, Imbert-Fernandez Y, JA C. Inhibition of glucose metabolism through treatment of BRAF mutated metastatic melanoma with Vemurafenib. J Clin Oncol. 2019;37(15 supp).

[28] Xu Y, Hao X, Ren Y, Xu Q, Liu X, Song S, et al. Research progress of abnormal lactate metabolism and lactate modification in immunotherapy of hepatocellular carcinoma. Front Oncol. 2022;12:1063423. 36686771 10.3389/fonc.2022.1063423 PMC9853001

[29] Siska PJ, Singer K, Evert K, Renner K, Kreutz M. The immunological Warburg effect: can a metabolic-tumor-stroma score (MeTS) guide cancer immunotherapy? Immunol Rev. 2020;295(1):187–202. 32157706 10.1111/imr.12846

[30] Ascierto ML, McMiller TL, Berger AE, Danilova L, Anders RA, Netto GJ, et al. The intratumoral balance between metabolic and Immunologic gene expression is associated with Anti-PD-1 response in patients with renal cell carcinoma. Cancer Immunol Res. 2016;4(9):726–33. 27491898 10.1158/2326-6066.CIR-16-0072 PMC5584610

[31] Harel M, Ortenberg R, Varanasi SK, Mangalhara KC, Mardamshina M, Markovits E, et al. Proteomics of melanoma response to immunotherapy reveals mitochondrial dependence. Cell. 2019;179(1):236–50. e18. 31495571 10.1016/j.cell.2019.08.012 PMC7993352

[32] Daneshmandi S, Wegiel B, Seth P. Blockade of lactate Dehydrogenase-A (LDH-A) improves efficacy of Anti-Programmed cell Death-1 (PD-1) therapy in melanoma. Cancers (Basel). 2019;11(4). 10.3390/cancers11040450 PMC6521327 30934955

[33] Shimizu A, Kaira K, Kato M, Yasuda M, Takahashi A, Tominaga H, et al. Prognostic significance of L-type amino acid transporter 1 (LAT1) expression in cutaneous melanoma. Melanoma Res. 2015;25(5):399–405. 26237765 10.1097/CMR.0000000000000181

[34] Badawy AA. Tryptophan metabolism and disposition in cancer biology and immunotherapy. Biosci Rep. 2022;42(11). 10.1042/BSR20221682 PMC9653095 36286592

[35] Hubkova B, Valko-Rokytovska M, Cizmarova B, Zabavnikova M, Marekova M, Birkova A. Tryptophan: its metabolism along the kynurenine, serotonin, and Indole pathway in malignant melanoma. Int J Mol Sci. 2022;23(16). 10.3390/ijms23169160 PMC9408957 36012419

[36] Mitchell TC, Hamid O, Smith DC, Bauer TM, Wasser JS, Olszanski AJ, et al. Epacadostat plus pembrolizumab in patients with advanced solid tumors: phase I results from a multicenter, Open-Label phase I/II trial (ECHO-202/KEYNOTE-037). J Clin Oncol. 2018;36(32):3223–30. 30265610 10.1200/JCO.2018.78.9602 PMC6225502

[37] Long GV, Dummer R, Hamid O, Gajewski TF, Caglevic C, Dalle S, et al. Epacadostat plus pembrolizumab versus placebo plus pembrolizumab in patients with unresectable or metastatic melanoma (ECHO-301/KEYNOTE-252): a phase 3, randomised, double-blind study. Lancet Oncol. 2019;20(8):1083–97. 31221619 10.1016/S1470-2045(19)30274-8

[38] Slominski A, Semak I, Pisarchik A, Sweatman T, Szczesniewski A, Wortsman J. Conversion of L-tryptophan to serotonin and melatonin in human melanoma cells. FEBS Lett. 2002;511(1–3):102–6. 11821057 10.1016/s0014-5793(01)03319-1

[39] Oldan JD, Giglio BC, Smith E, Zhao W, Bouchard DM, Ivanovic M, et al. Increased Tryptophan, but not increased glucose metabolism, predict resistance of pembrolizumab in stage III/IV melanoma. Oncoimmunology. 2023;12(1):2204753. 37123046 10.1080/2162402X.2023.2204753 PMC10142396

[40] Azuma K, Xiang H, Tagami T, Kasajima R, Kato Y, Karakawa S et al. Clinical significance of plasma-free amino acids and Tryptophan metabolites in patients with non-small cell lung cancer receiving PD-1 inhibitor: a pilot cohort study for developing a prognostic multivariate model. J Immunother Cancer. 2022;10(5). 10.1136/jitc-2021-004420 PMC9109096 35569917

[41] Yan C, Wu D, Gan L, Wang J, Yang W, Xu B. Significant metabolic alterations in non-small cell lung cancer patients by epidermal growth factor receptor-targeted therapy and PD-1/PD-L1 immunotherapy. Front Pharmacol. 2022;13:949745. 36034789 10.3389/fphar.2022.949745 PMC9403486

[42] Niu YC, Feng RN, Hou Y, Li K, Kang Z, Wang J, et al. Histidine and arginine are associated with inflammation and oxidative stress in obese women. Br J Nutr. 2012;108(1):57–61. 21996294 10.1017/S0007114511005289

[43] Uchiyama K, Yagi N, Mizushima K, Higashimura Y, Hirai Y, Okayama T, et al. Serum metabolomics analysis for early detection of colorectal cancer. J Gastroenterol. 2017;52(6):677–94. 27650200 10.1007/s00535-016-1261-6

[44] Taylor NJ, Gaynanova I, Eschrich SA, Welsh EA, Garrett TJ, Beecher C, et al. Metabolomics of primary cutaneous melanoma and matched adjacent extratumoral microenvironment. PLoS ONE. 2020;15(10):e0240849. 33108391 10.1371/journal.pone.0240849 PMC7591037

[45] Nie X, Xia L, Gao F, Liu L, Yang Y, Chen Y, et al. Serum metabolite biomarkers predictive of response to PD-1 Blockade therapy in Non-Small cell lung cancer. Front Mol Biosci. 2021;8:678753. 34095230 10.3389/fmolb.2021.678753 PMC8176105

[46] Zhang H, Liu J, Yuan W, Zhang Q, Luo X, Li Y, et al. Ammonia-induced lysosomal and mitochondrial damage causes cell death of effector CD8(+) T cells. Nat Cell Biol. 2024;26(11):1892–902. 39261719 10.1038/s41556-024-01503-x

[47] Li Z, Lin J, Yin P. Ammonia death: a novel potential strategy to augment immunotherapy in cancer. Cancer Gene Ther. 2024;31(12):1751–3. 39528819 10.1038/s41417-024-00851-y

