# Supplementary material for: Metabolomic signatures in liquid biopsy are associated with overall survival in metastatic melanoma patients treated with immune checkpoint inhibitor therapy
Source: J Exp Clin Cancer Res. 2025 Apr 10;44:119. doi: 10.1186/s13046-025-03378-8 (PMC11983745; doi:10.1186/s13046-025-03378-8)
Supplement: Supplementary file 1 — Supplementary Material 1 [file 13046_2025_3378_MOESM1_ESM.docx]

**Table S1.** Enriched molecular function analysis on significant metabolites in the sera of metastatic melanoma patients treated with immunotherapy at first line.

| **Pathway Name** | **Metabolites** | **p** |
| --- | --- | --- |
| Biotin metabolism | Lysine, ATP | 0.00243 |
| Ammonia recycling | Asparagine, histidine, ATP | 0.00278 |
| Lactose degradation | Glucose, ATP | 0.00311 |
| Gluconeogenesis | Glucose, lactate, ATP | 0.00334 |
| Trehalose degradation | Glucose, ATP | 0.0047 |
| Spermidine and Spermine Biosynthesis | Ornithine, ATP | 0.0126 |
| Lactose synthesis | Glucose, ATP | 0.014 |
| Warburg effect | Glucose, lactate, ATP | 0.0158 |
| Tryptophan metabolism | ATP, formate, tryptophan | 0.0173 |
| Transfer of acetyl groups into mitochondria | Glucose, ATP | 0.0186 |
| Glycolysis | Glucose, ATP | 0.0203 |
| Urea cycle | ATP, ornithine | 0.0295 |
| Folate metabolism | ATP, formate | 0.0315 |
| Methylhistidine metabolism | Histidine | 0.0394 |
| Aspartate metabolism | Asparagine, ATP | 0.0447 |

**Table S2.** Enriched molecular function analysis on significant metabolites in the sera of metastatic melanoma patients treated with ipilimumab at first line.

| **Pathway Name** | **Metabolites** | **p** |
| --- | --- | --- |
| Ammonia recycling | Asparagine, histidine, aspartate, ATP | 0.00014 |
| Glutamate metabolism | Glutathione, alanine, aspartate, ATP | 0.00079 |
| Glutathione metabolism | Glutatione, alanine, ATP | 0.00082 |
| Urea cycle | Alanine, aspartate, ATP | 0.0022 |
| Lactose degradation | Glucose, ATP | 0.0030 |
| Aspartate metabolism | Asparagine, aspartate, ATP | 0.0037 |
| Glucose-alanine cycle | Glucose, alanine | 0.0063 |
| Alanine metabolism | Alanine, ATP | 0.011 |
| Lactose synthesis | Glucose, ATP | 0.015 |
| Valine, leucine and isoleucine degradation | Isoleucine, ATP, valine | 0.017 |
| Transfer of acetyl groups into mitochondria | Glucose, ATP | 0.018 |
| Glycolysis | Glucose, ATP | 0.023 |
| Phenylalanine and tyrosine metabolism | Phenylalanine, ATP | 0.028 |
| Selenoamino acid metabolism | Alanine, ATP | 0.028 |
| Methylhistidine metabolism | Histidine | 0.038 |
| Beta-alanine metabolism | Histidine, aspartate | 0.041 |

**Table S3.** Enriched molecular function analysis on significant metabolites in the sera of metastatic melanoma patients treated with nivolumab at first line.

| **Pathway Name** | **Metabolites** | **p** |
| --- | --- | --- |
| Urea cycle | Glutamate, ornithine, glutamine | 0.0015 |
| Ammonia recycling | Glutamate, histidine, glutamine | 0.0020 |
| Glucose-alanine cycle | Glutamate, glucose | 0.0051 |
| Arginine and proline metabolism | Glutamate, proline, ornithine | 0.0088 |
| Warburg effect | Glucose, glutamate, glutamine | 0.011 |
| Valine, leucine and isoleucine degradation | Glutamate, isoleucine, valine | 0.012 |
| Lysine degradation | Glutamate, lysine | 0.026 |
| Amino sugar metabolism | Glutamate, glutamine | 0.032 |
| Beta-alanine metabolism | Glutamate, histidine | 0.033 |
| Methylhistidine metabolism | Histidine | 0.035 |
| Aspartate metabolism | Glutamate, glutamine | 0.035 |
| Nicotinate and nicotinamide metabolism | Glutamate, glutamine | 0.039 |
| Propanoate metabolism | Glutamate, valine | 0.049 |

**Table S4.** Enriched molecular function analysis on significant metabolites in the sera of metastatic melanoma patients treated with ipilimumab plus nivolumab at first line.

| **Pathway Name** | **Metabolites** | **p** |
| --- | --- | --- |
| Tryptophan metabolism | Formate, glutamate, tryptophan | 0.0017 |
| Folate metabolism | Formate, glutamate | 0.030 |
| Lysine degradation | Lysine, glutamate | 0.032 |
| Ammonia recycling | Glutamate, histidine | 0.036 |
| Methylhistidine metabolism | Histidine | 0.039 |
| Beta-alanine metabolism | Glutamate, histidine | 0.041 |
