# Supplementary material for: Metabolomic signatures in liquid biopsy are associated with overall survival in metastatic melanoma patients treated with immune checkpoint inhibitor therapy
Source: J Exp Clin Cancer Res. 2025 Apr 10;44:119. doi: 10.1186/s13046-025-03378-8 (PMC11983745; doi:10.1186/s13046-025-03378-8)
Supplement: Supplementary file 2 — Supplementary Material 2 [file 13046_2025_3378_MOESM2_ESM.docx]

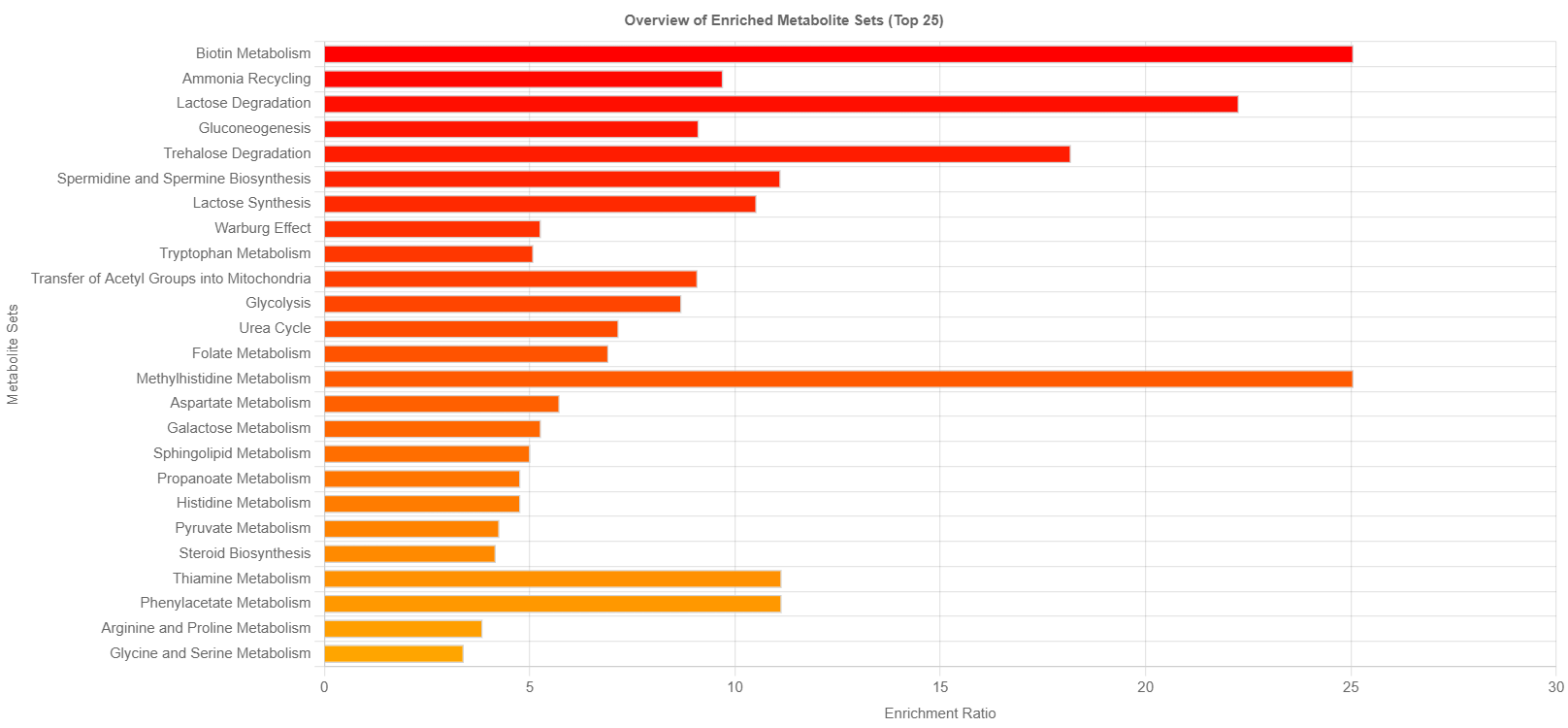


**Fig. S1.** The most significant pathways, in which significant metabolites in the metabolome of the sera of metastatic melanoma patients treated with immunotherapy at first-line, are reported by bars using colors, from yellow to red, to indicate increasing levels of statistically significance (p-values from the pathway enrichment analysis reported in Table S1).

**A**


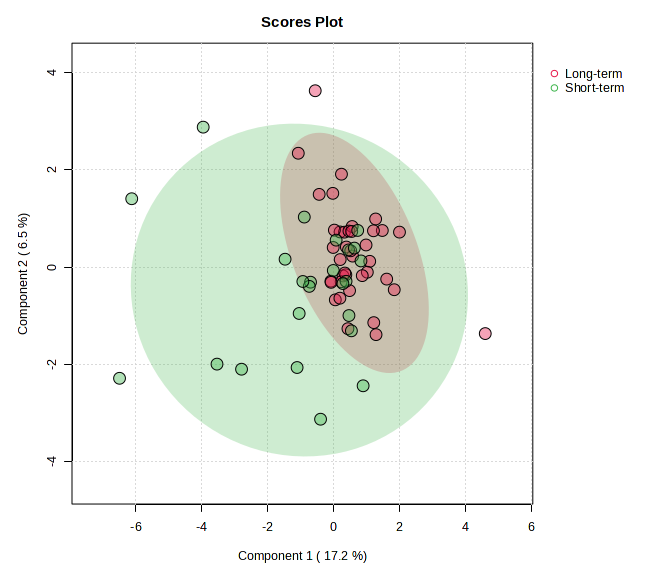


**B**


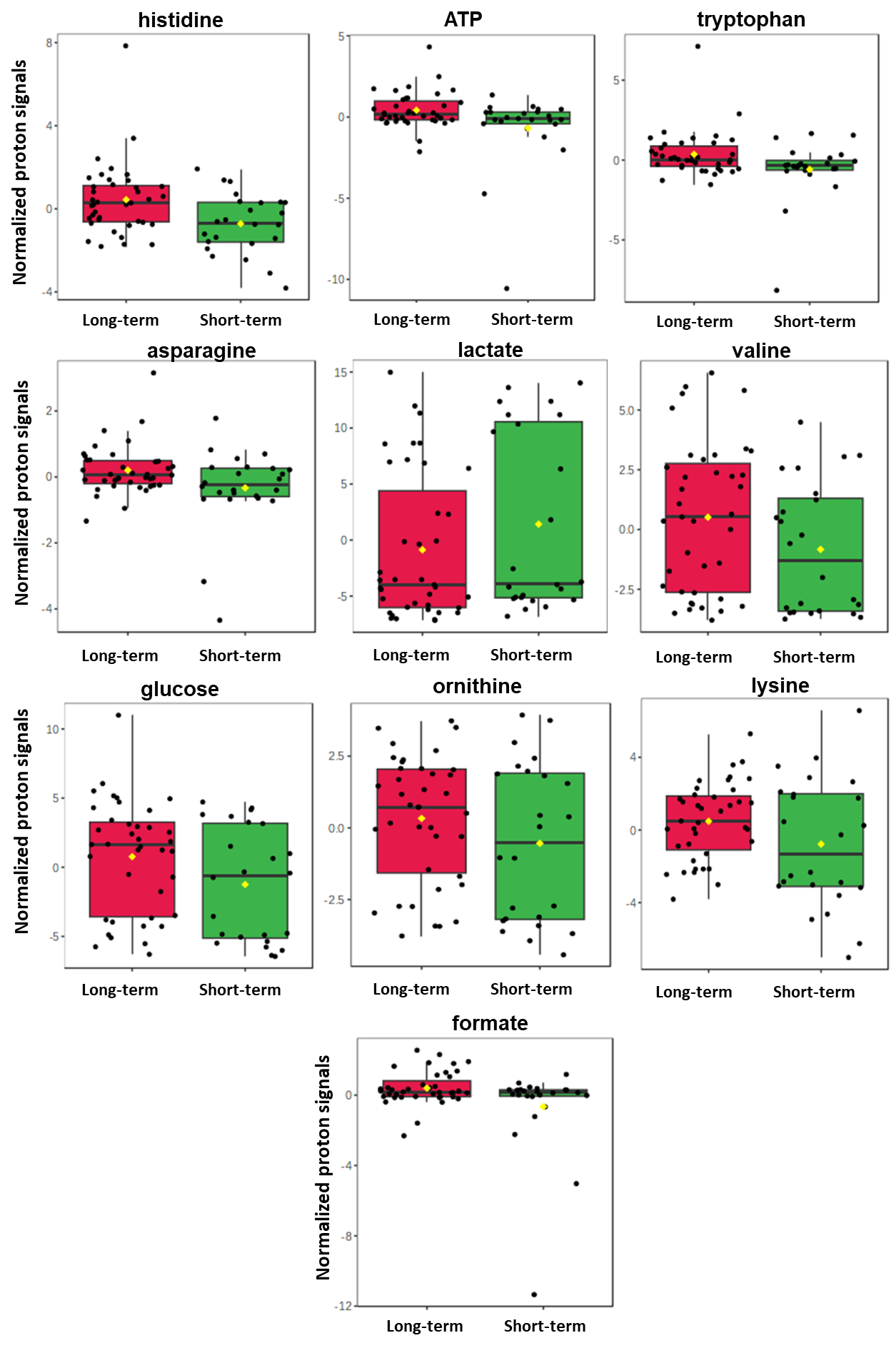


**Fig. S2.** **A)** Score plot related to metabolomic profiling of the sera of metastatic melanoma patients treated with immunotherapy at first-line enrolled in validation set. **B)** Box-and whisker plot of the normalized proton signals of the selected metabolites reported in Figure1B analyzed in Long-term and Short-term Groups from the validation set.


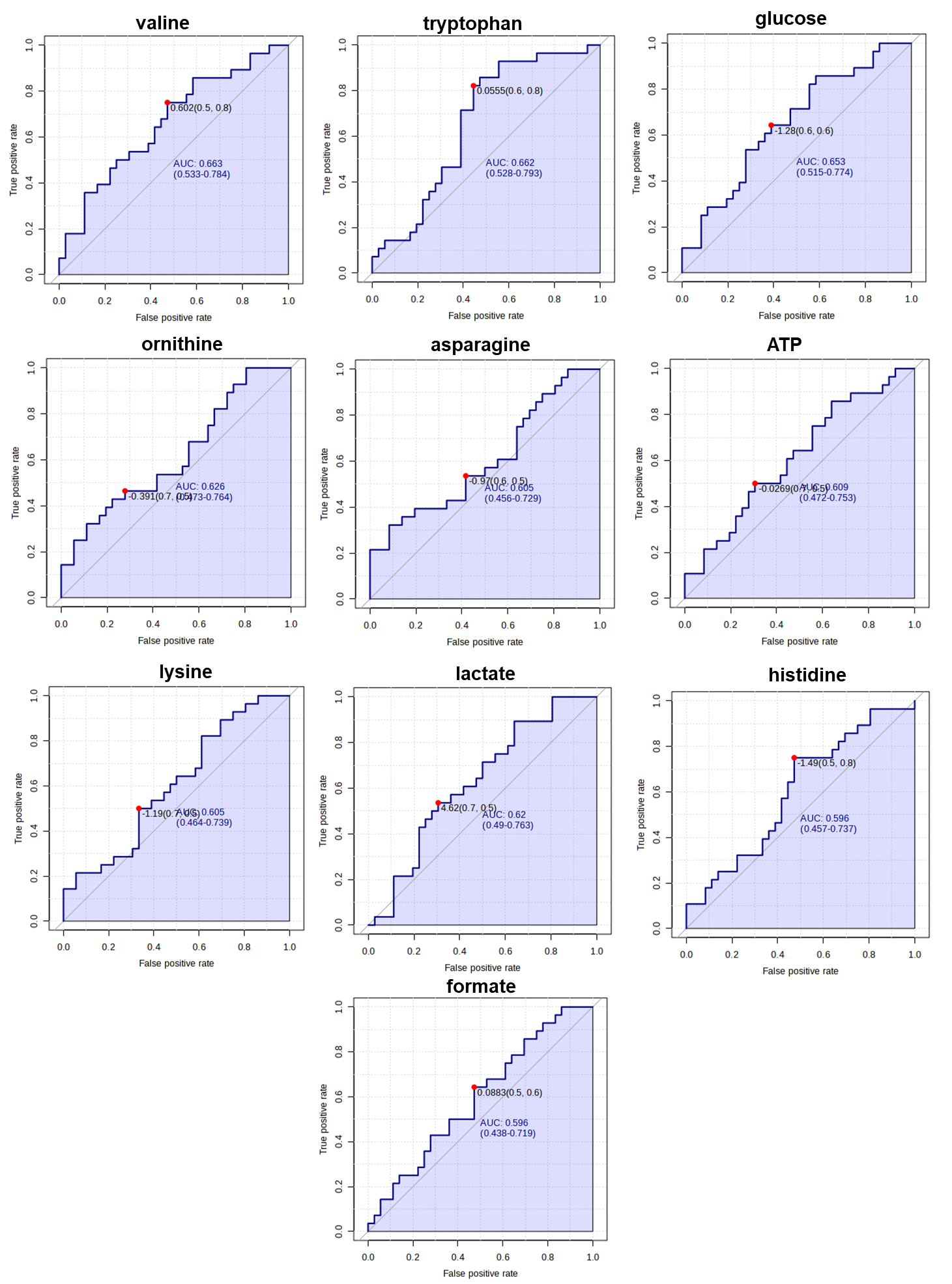


**Fig. S3**. ROC curves performed on significant metabolites in the sera of metastatic melanoma patients treated with immunotherapy at first-line, selected by sPLS-DA. The cutoff values are evidenced by red circles.


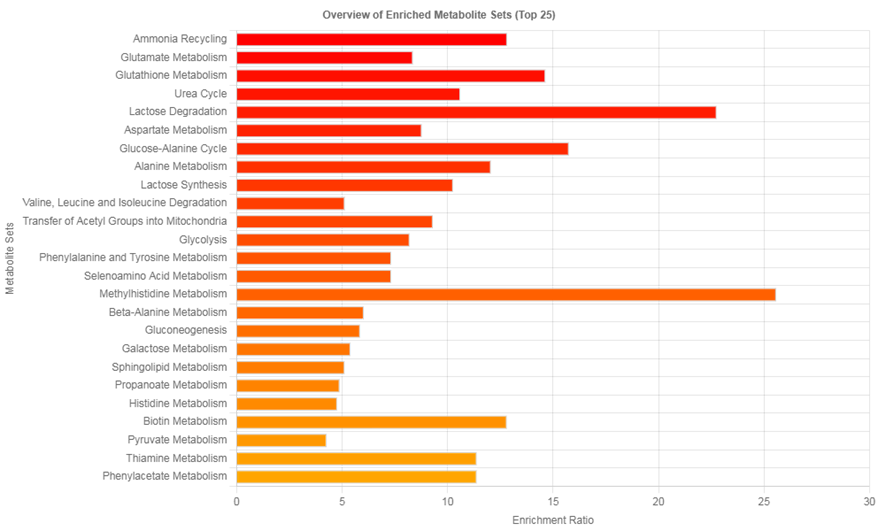


**Fig. S4.** The most significant pathways, in which significant metabolites in the sera of metastatic melanoma patients treated with ipilimumab at first-line, are reported by bars using colors, from yellow to red, to indicate increasing levels of statistically significance (p-values from the pathway enrichment analysis reported in Table S2).

**A**

**
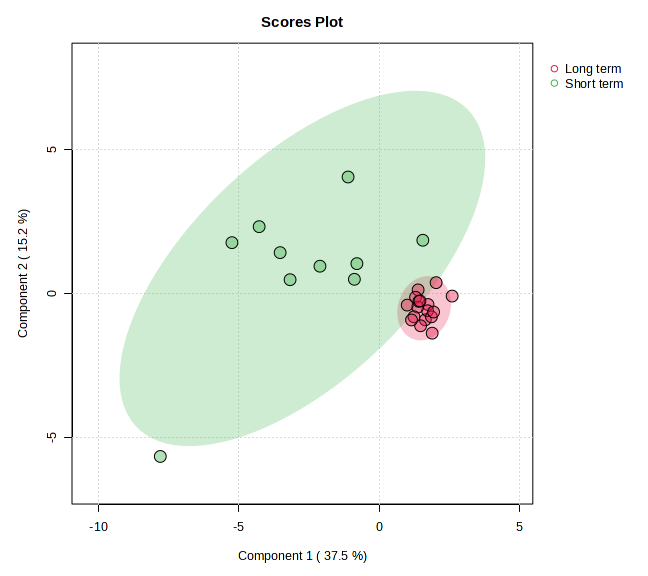
**

**B**


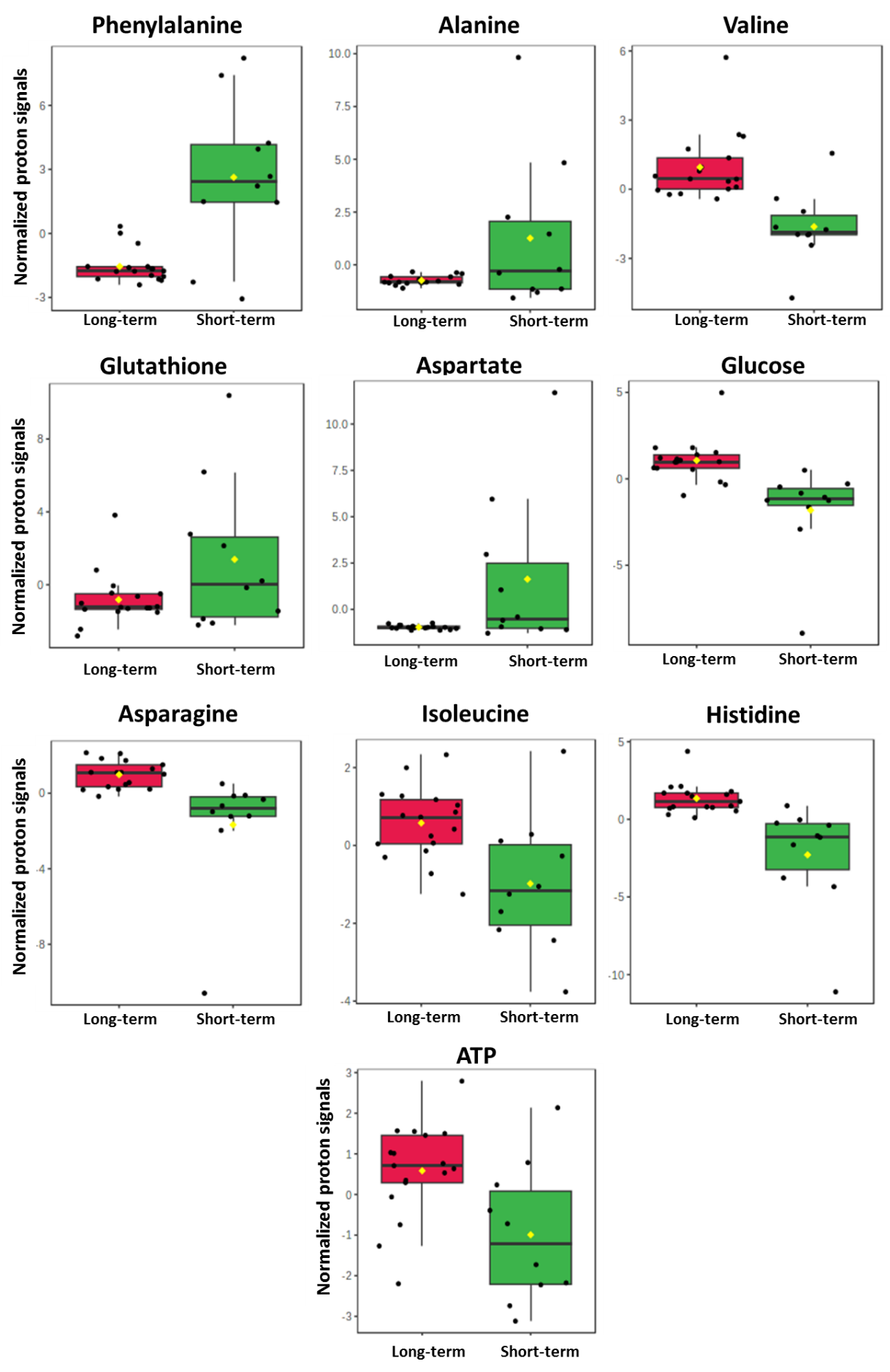


**Fig. S5.** **A)** Score plot related to metabolomic profiling of the sera of metastatic melanoma patients treated with ipilimumab at first-line enrolled in validation set. **B)** Box-and-whisker plot of the normalized proton signals of the selected metabolites reported in Figure3B analyzed in Long-term and Short-term Groups from the validation set.


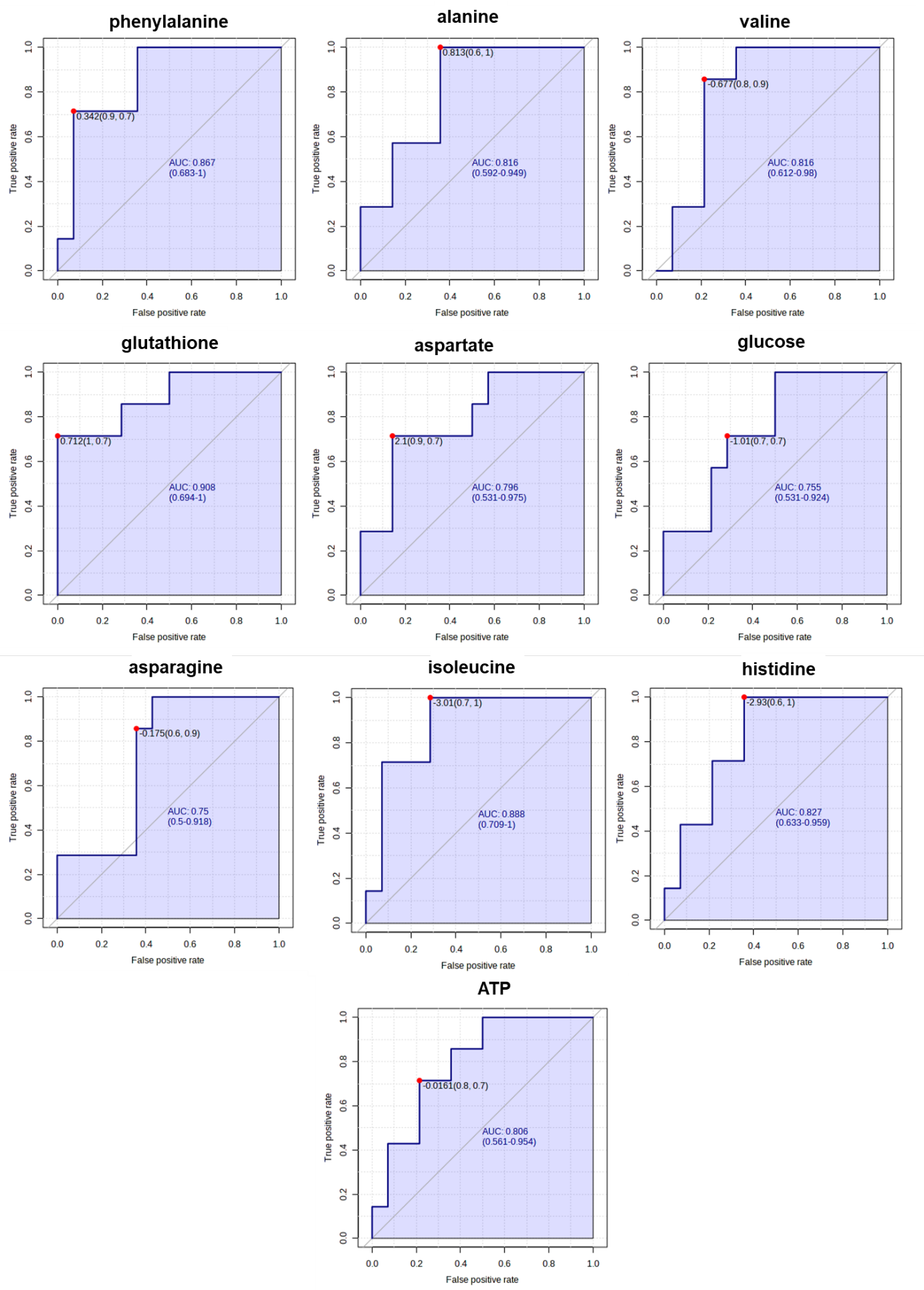


**Fig. S6**. ROC curves performed on significant metabolites in the sera of metastatic melanoma patients treated with ipilimumab at first-line, selected by sPLS-DA. The cutoff values are evidenced by red circles.


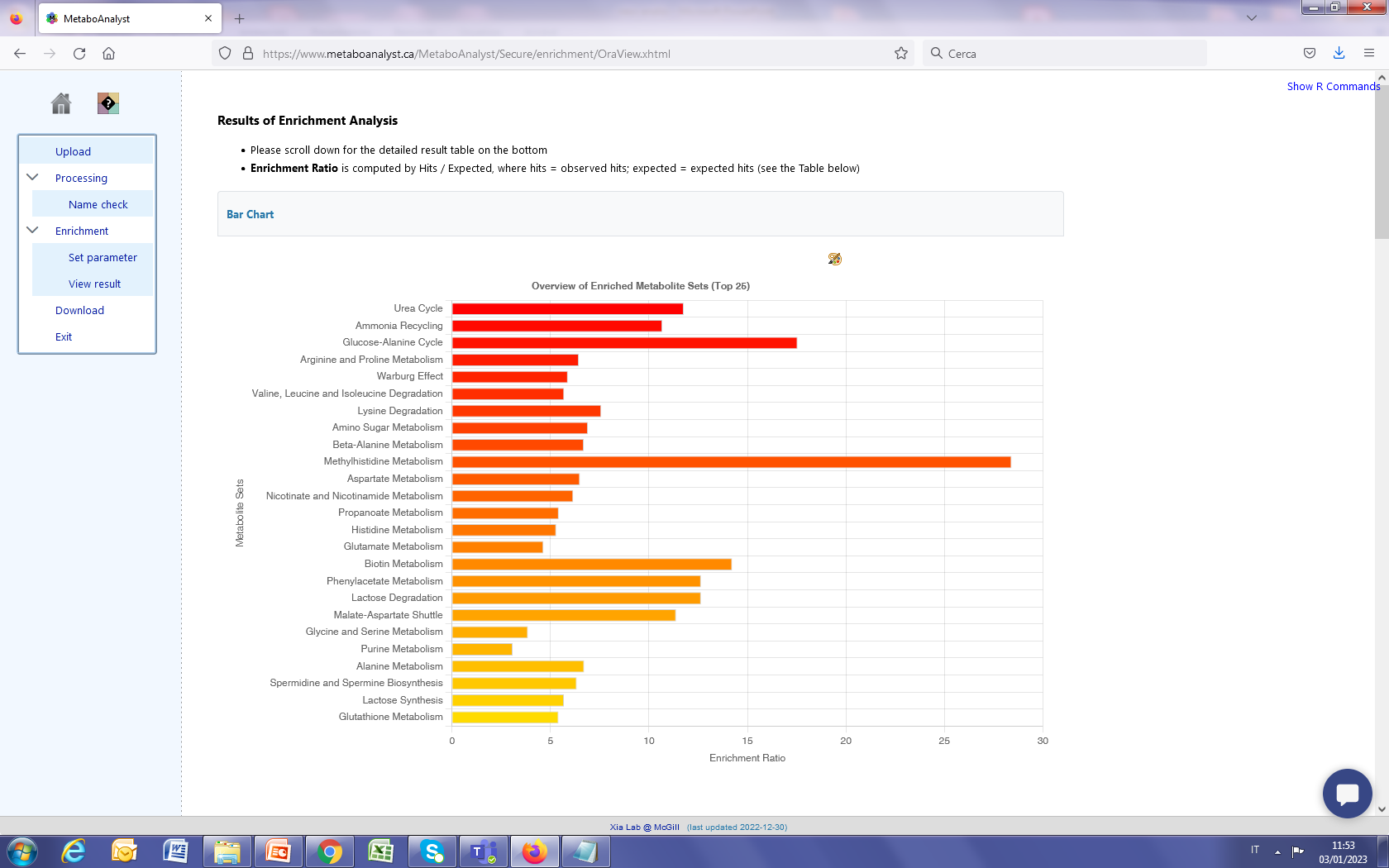


**Fig. S7.** The most significant pathways, in which significant metabolites in the sera of metastatic melanoma patients treated with nivolumab at first-line, are reported by bars using colors, from yellow to red, to indicate increasing levels of statistically significance (p-values from the pathway enrichment analysis reported in Table S3).

**A**

**
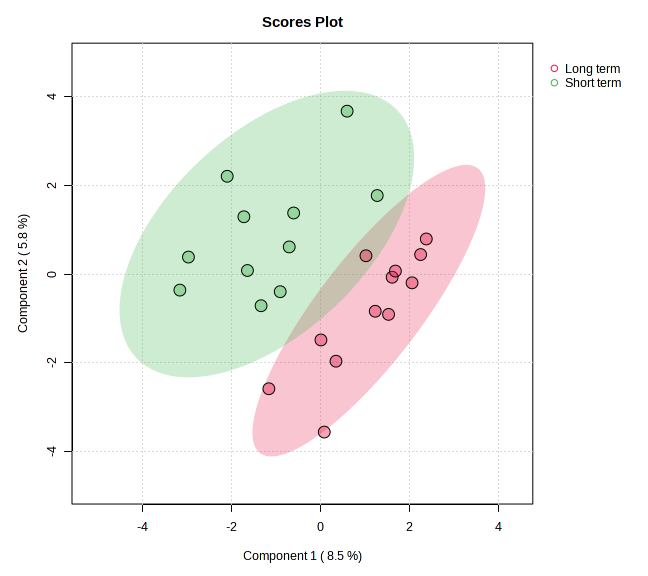
**

**B**

**
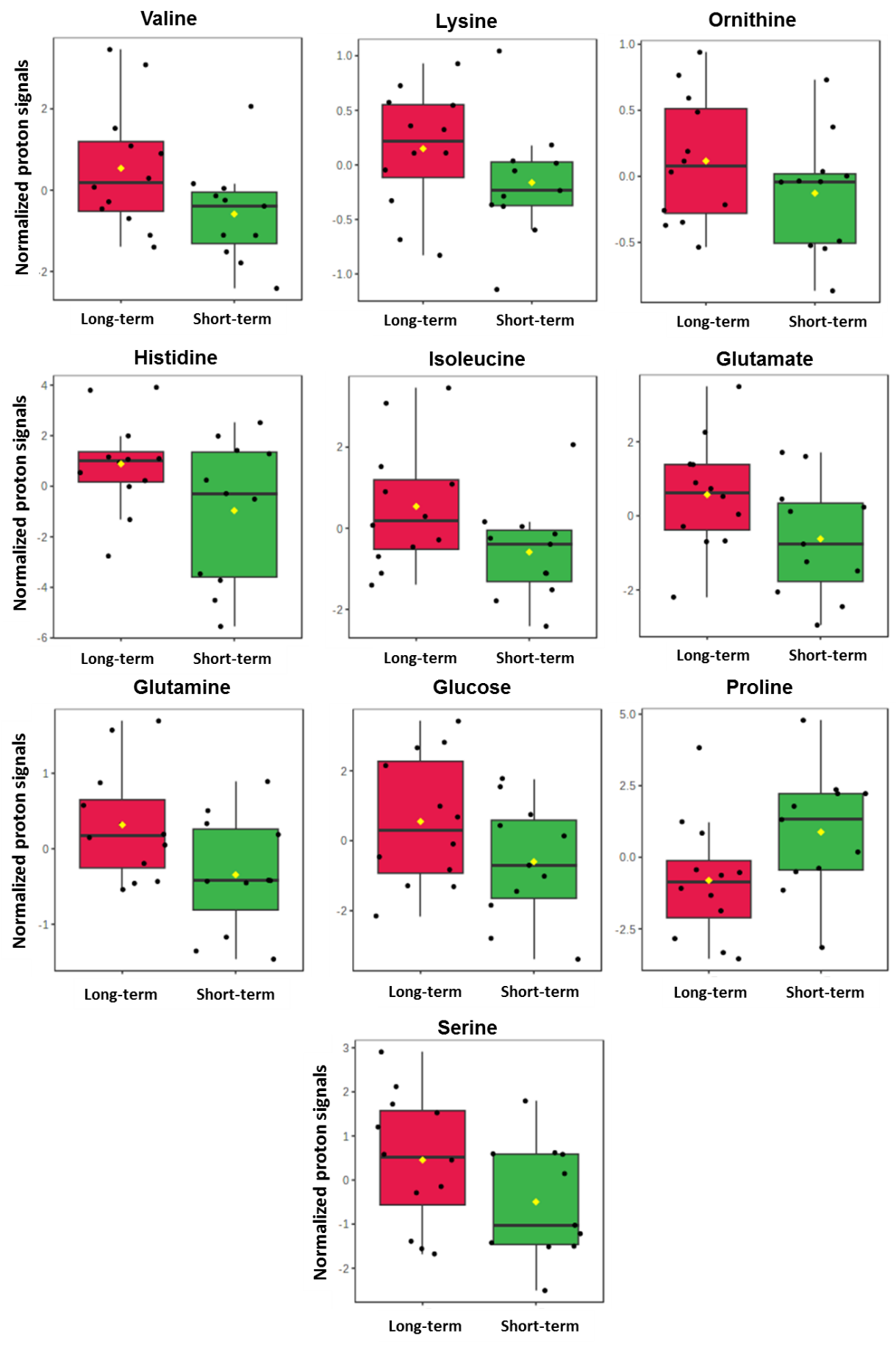
**

**Fig. S8.** **A)** Score plot related to metabolomic profiling of the sera of metastatic melanoma patients treated with nivolumab at first-line enrolled in validation set. **B)** Box-and whisker plot of the normalized proton signals of the selected metabolites reported in Figure5B analyzed in Long-term and Short-term Groups from the validation set.


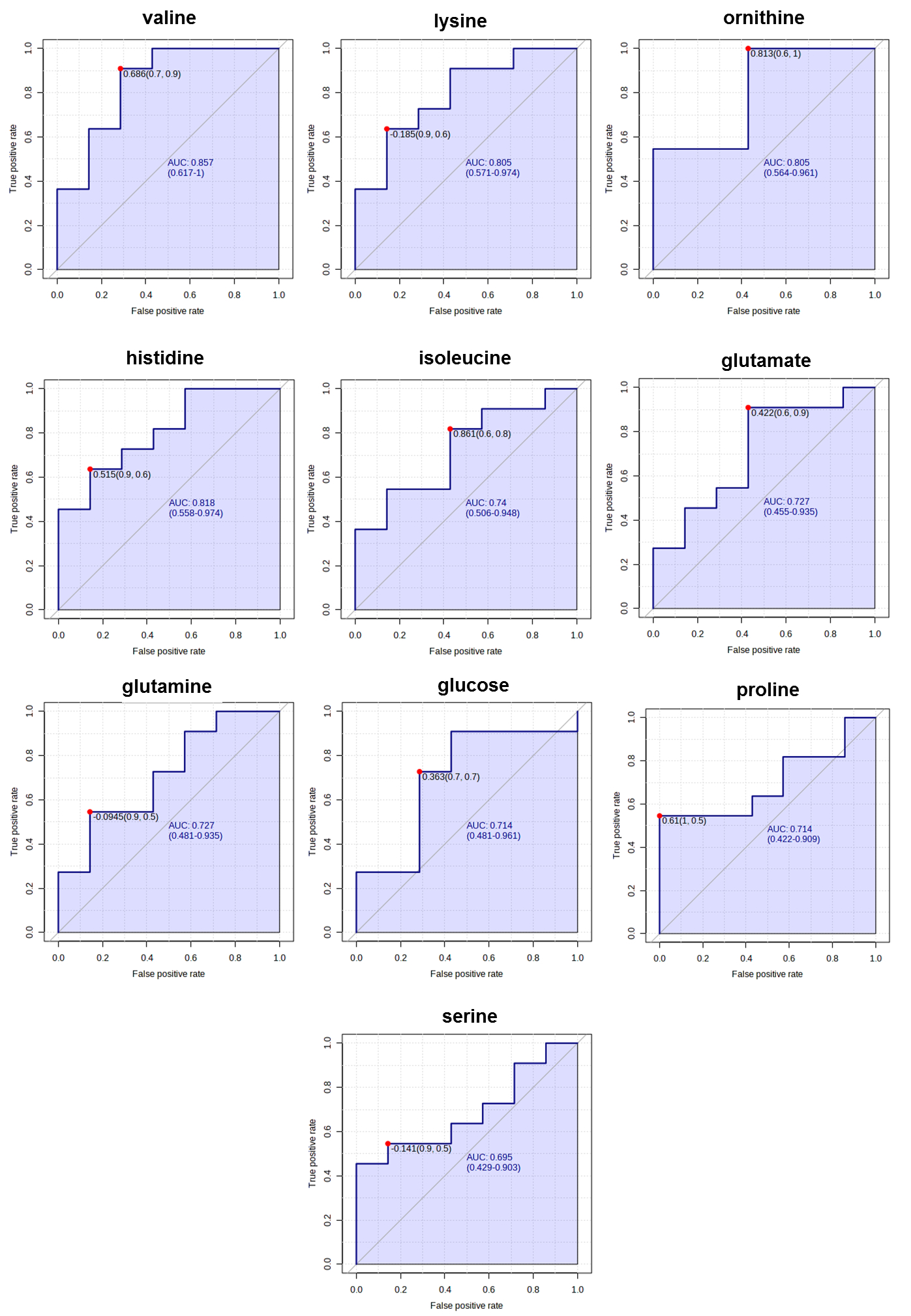


**Fig. S9**. ROC curves performed on significant metabolites in the sera of metastatic melanoma patients treated with nivolumab at first-line, selected by sPLS-DA. The cutoff values are evidenced by red circles.


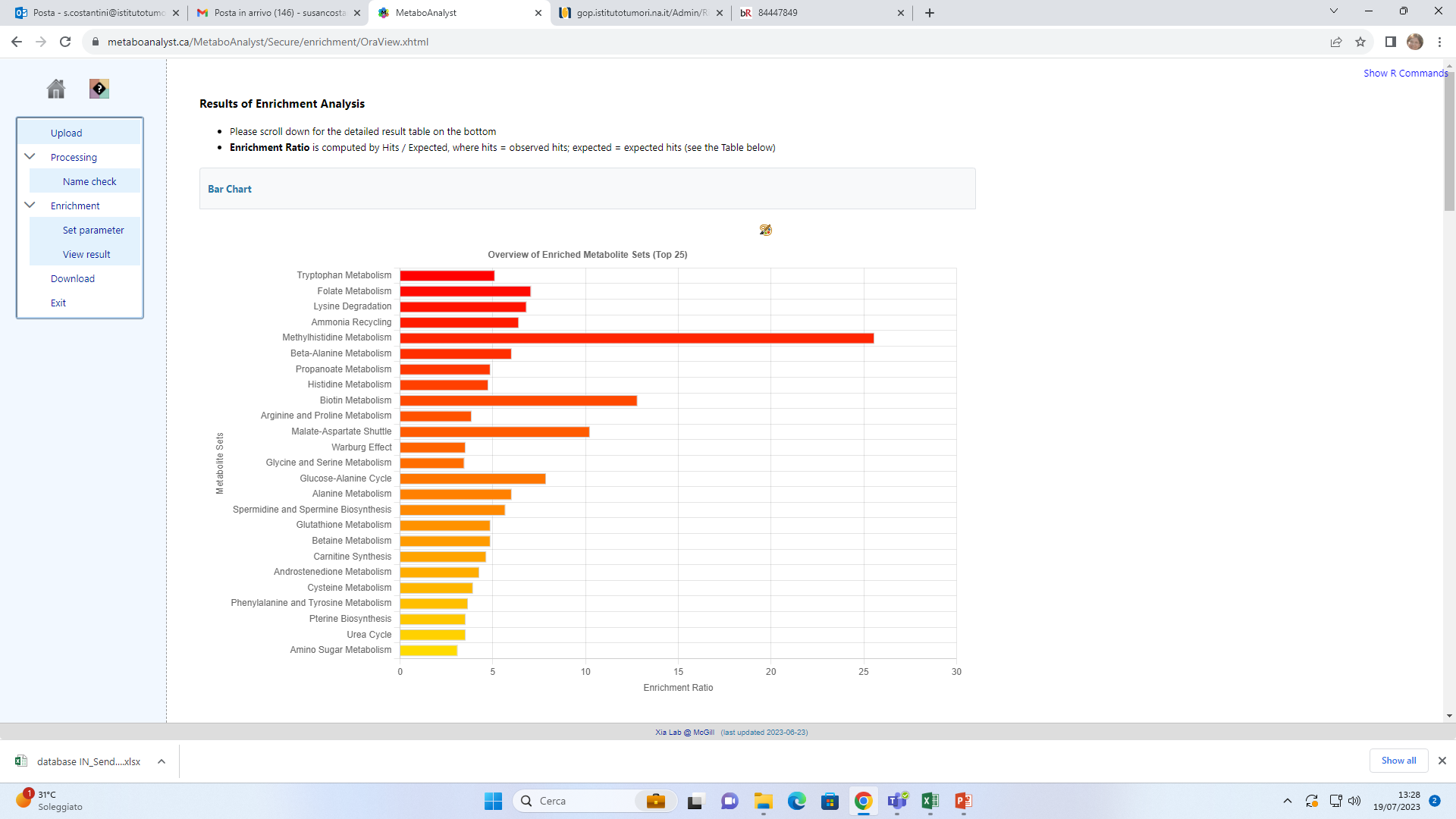


**Fig. S10.** The most significant pathways, in which significant metabolites in the sera of metastatic melanoma patients treated with ipilimumab plus nivolumab at first-line, are reported by bars using colors, from yellow to red, to indicate increasing levels of statistically significance (p values from the pathway enrichment analysis reported in Table S4).

**A**

**
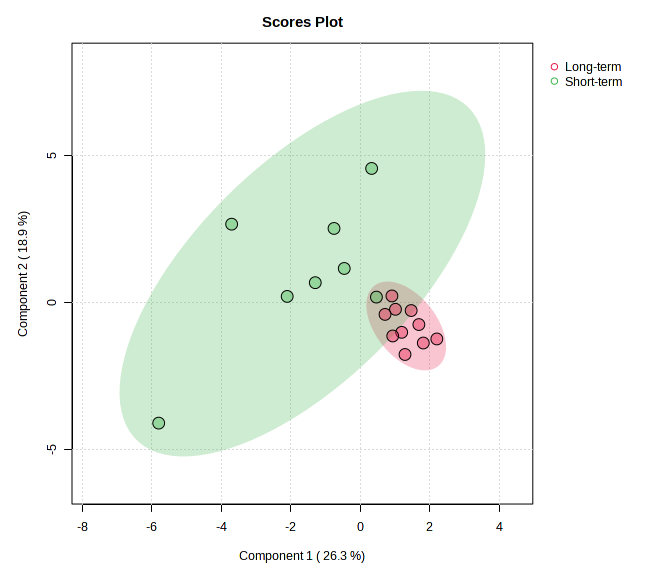
**

**B**

**
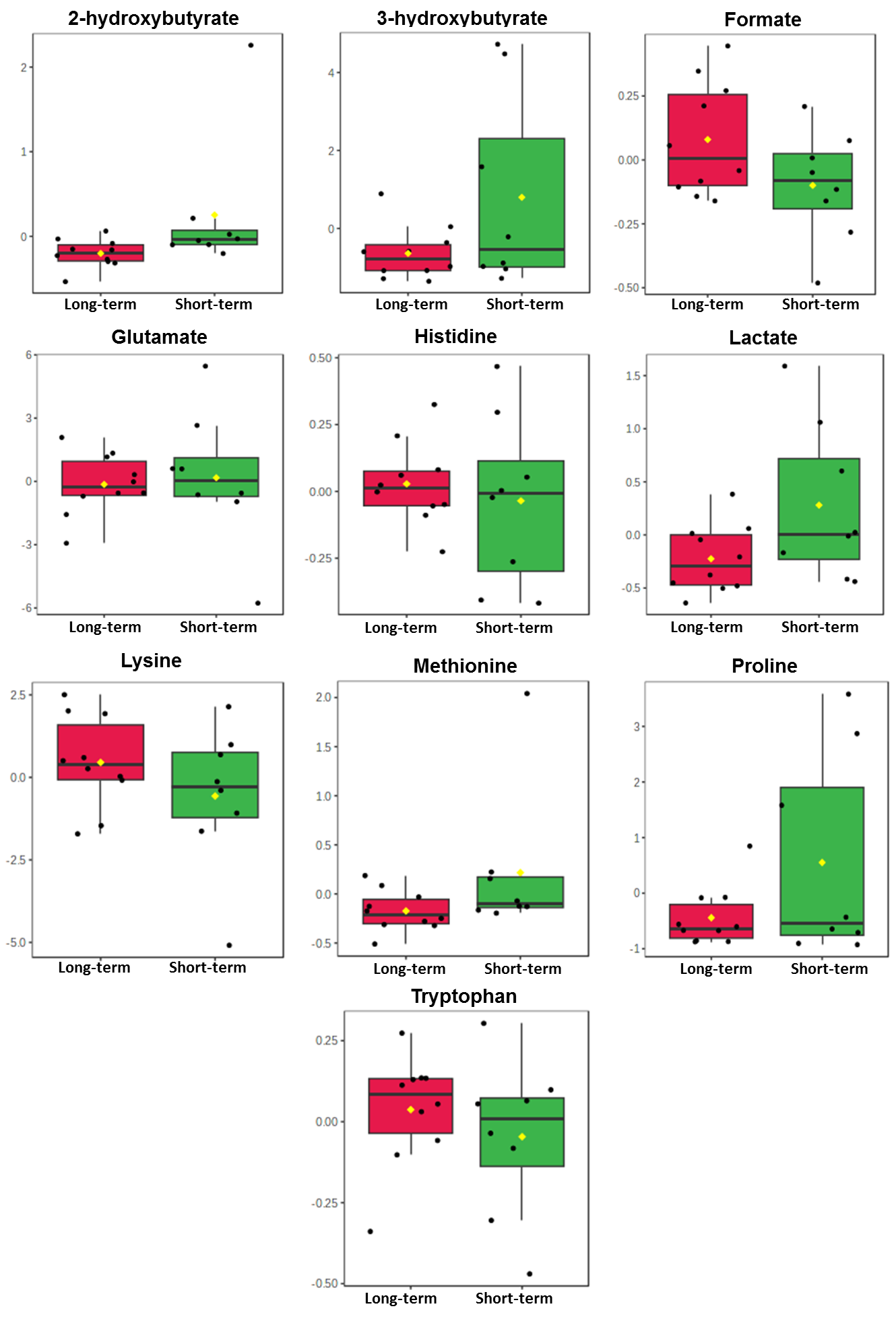
**

**Fig. S11.** **A)** Score plot related to metabolomic profiling of the sera of metastatic melanoma patients treated with ipilimumab plus nivolumab at first-line enrolled in validation set. **B)** Box-and whisker plot of the normalized proton signals of the selected metabolites reported in Figure7B analyzed in Long-term and Short-term Groups from the validation set.

**
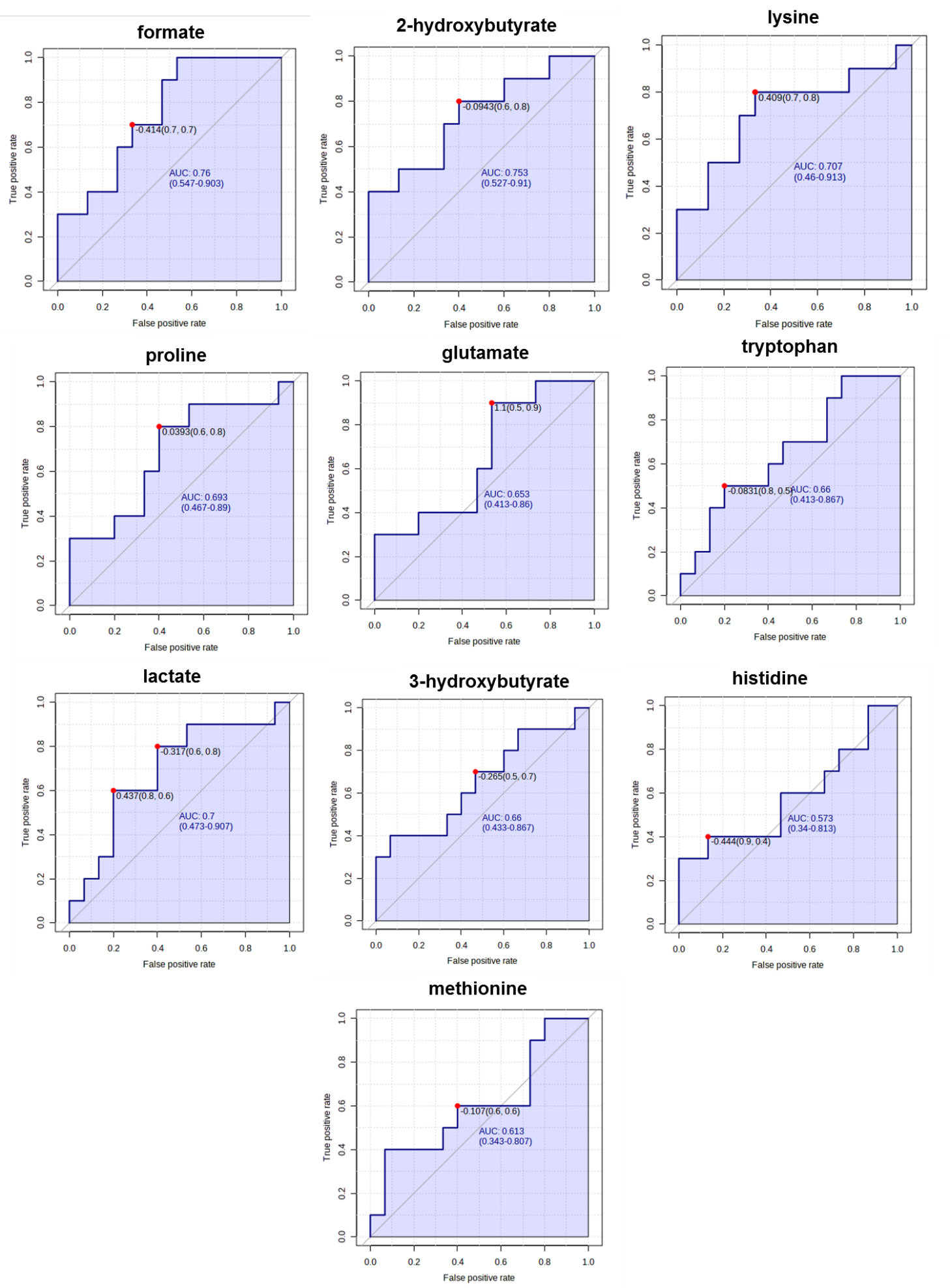
**

**Fig. S12**. ROC curves performed on significant metabolites in the sera of metastatic melanoma patients treated with ipilimumab plus nivolumab at first-line, selected by sPLS-DA. The cutoff values are evidenced by red circles.


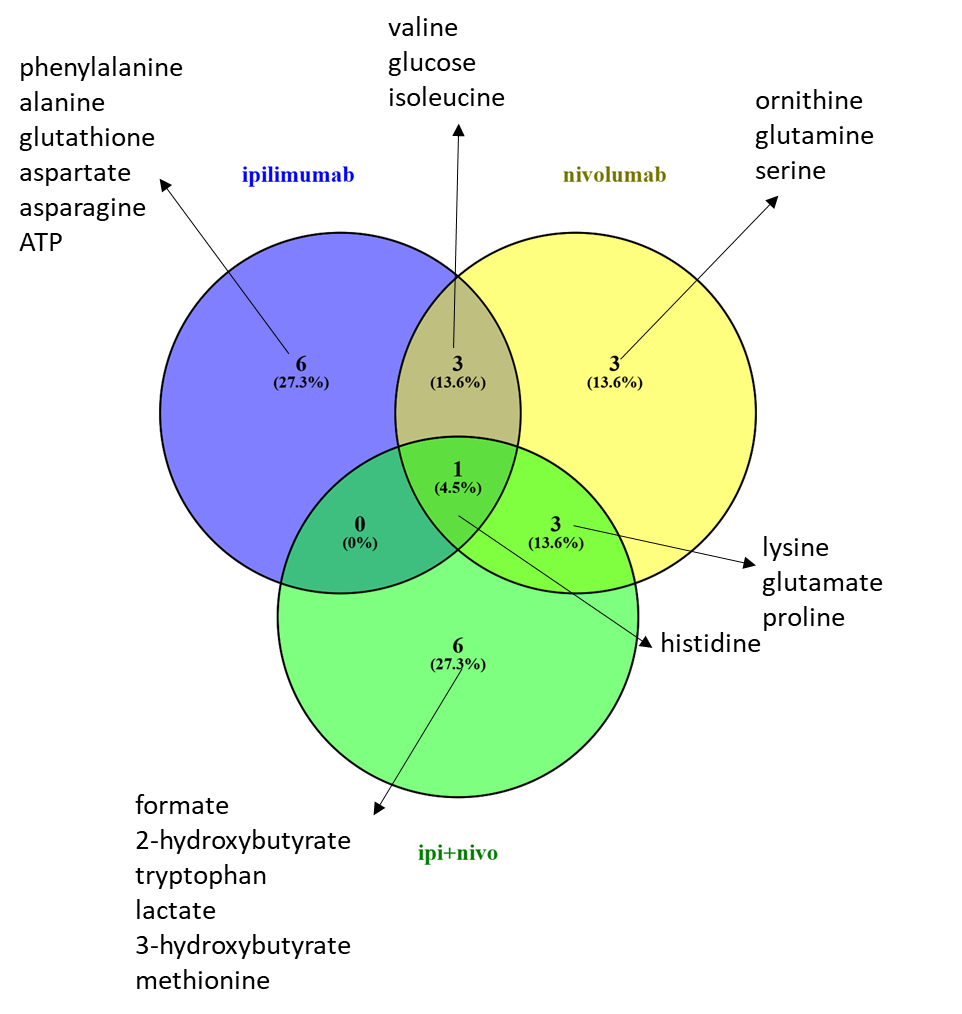


**Fig. S13.** Venn diagram performed to identify the common significant metabolites in three different patients’ sub-groups.


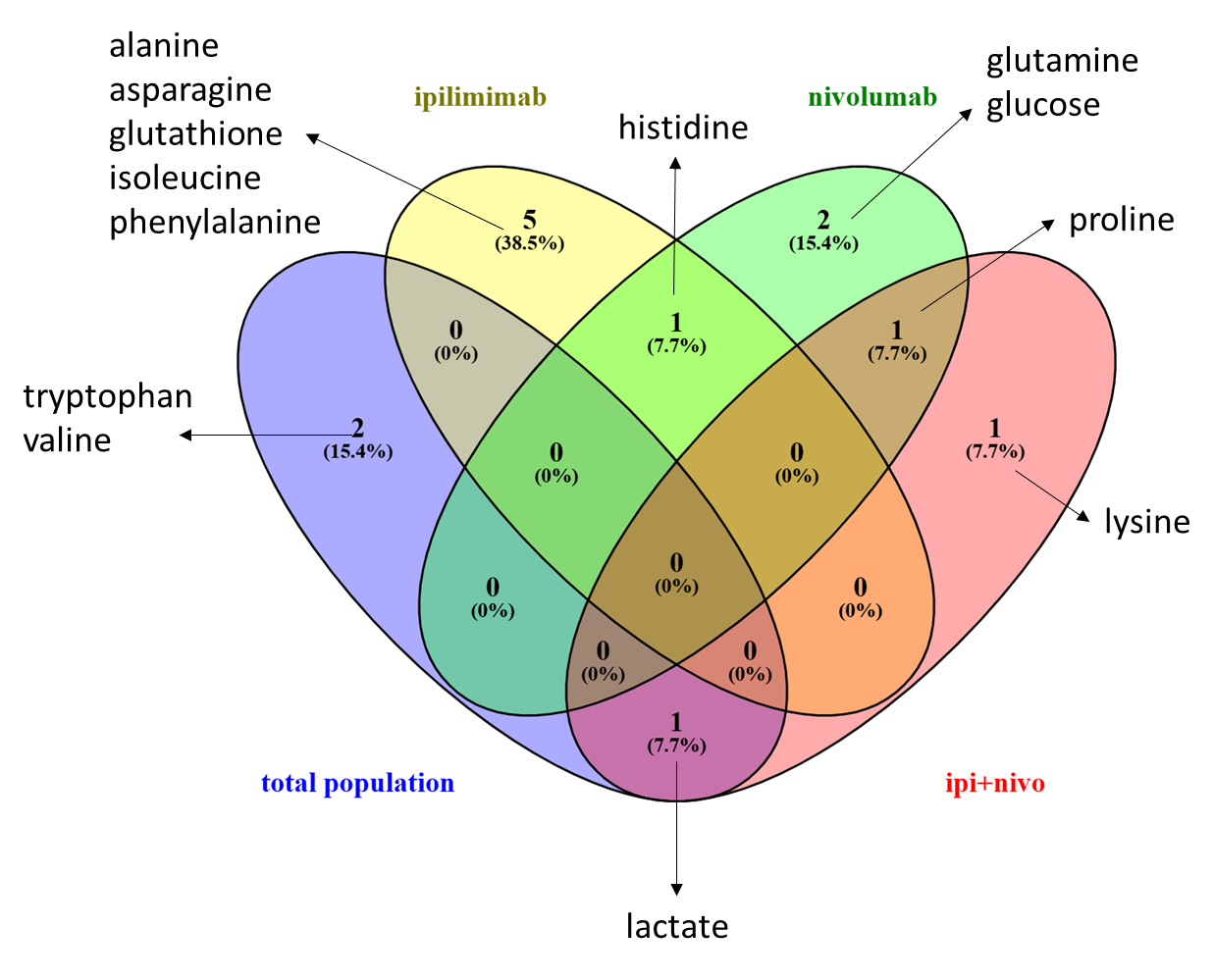


**Fig. S14.** Venn diagram performed to identify the common significant metabolites among those that predict OS after univariate analysis in the total population and three different sub-groups.
